# Classification of the mitochondrial ribosomal protein-associated molecular subtypes and identified a serological diagnostic biomarker in hepatocellular carcinoma

**DOI:** 10.3389/fsurg.2022.1062659

**Published:** 2023-01-06

**Authors:** Cong Xie, Juan Hu, Qin Hu, Linshan Jiang, Weixian Chen

**Affiliations:** Department of Laboratory Medicine, The Second Affiliated Hospital of Chongqing Medical University, Chongqing, China

**Keywords:** mitochondrial ribosomal protein, molecular subtype, mrpl9, hepatocellular carcinoma, biomarker

## Abstract

**Purpose:**

The objective of this study was to sort out innovative molecular subtypes associated with mitochondrial ribosomal proteins (MRPs) to predict clinical therapy response and determine the presence of circulating markers in hepatocellular carcinoma (HCC) patients.

**Methods:**

Using an unsupervised clustering method, we categorized the relative molecular subtypes of MRPs in HCC patients. The prognosis, biological properties, immune checkpoint inhibitor and chemotherapy response of the patients were clarified. A signature and nomogram were developed to evaluate the prognosis. Enzyme-linked immunosorbent assay (ELISA) measured serum mitochondrial ribosomal protein L9 (MRPL9) levels in liver disease patients and normal individuals. Receiver operating characteristic (ROC) curves were conducted to calculate the diagnostic effect. The Cell Counting Kit 8 was carried out to examine cell proliferation, and flow cytometry was used to investigate the cell cycle. Transwell assay was applied to investigate the potential of cell migration and invasion. Western blot detected corresponding changes of biological markers.

**Results:**

Participants were classified into two subtypes according to MRPs expression levels, which were characterized by different prognoses, biological features, and marked differences in response to chemotherapy and immune checkpoint inhibitors. Serum MRPL9 was significantly higher in HCC patients than in normal individuals and the benign liver disease group. ROC curve analysis showed that MRPL9 was superior to AFP and Ferritin in differentiating HCC from healthy and benign patients, or alone. Overexpressed MRPL9 could enhance aggressiveness and facilitate the G1/S progression in HCC cells.

**Conclusion:**

We constructed novel molecular subtypes based on MRPs expression in HCC patients, which provided valuable strategies for the prediction of prognosis and clinical personalized treatment. MRPL9 might act as a reliable circulating diagnostic biomarker and therapeutic target for HCC patients.

## Introduction

Primary liver cancer is the sixth most widely diagnosed cancer and the third major cause of tumor death worldwide in 2020, with approximately 906,000 new cases and 830,000 deaths (1). As the main histological type of liver cancer, HCC occupies the great majority of liver cancer diagnoses and deaths owing to the low early diagnosis rate, accelerated progress, invalid treatments for advanced stage, and a lack of early prognostic indicators. Current conventional HCC diagnostic markers are easily measured, such as alpha-fetoprotein (AFP) and ferritin (2). However, their clinical worth remains contentious because of their low specificity and sensitivity. Therefore, finding new biomarkers remains critical for the prognosis and diagnosis of HCC.

MRPs are nuclear genes that are synthesized in the cytoplasm and then shipped to the mitochondria for assembly. Increasing evidence confirmed that MRPs are involved not only in mitochondria oxidation phosphorylation (OXPHOS), but also in the regulation of cellular status, cell cycle, and mitochondria homeostasis (3, 4). Recently, several members of the MRP family were reported to be correlated with tumor progression. For example, it has been shown that MRPL15 is associated with the development of epithelial and non-small cell carcinoma (5, 6). MRPL41 interacts with mitochondrion-dependent mitochondria and is associated with p53 and p27 (Kip1) inhibitors (7). Furthermore, MRPS12 may act as a potential oncogene and may be a promising candidate for the prognosis of ovarian cancer (8).

MRPL9 is a component of the 39S subunit of the mitochondrial ribosome and is located on chromosome 19q13.2. The inferred amino acid sequence defines MRPL9 as a 27.5 kDa base protein with a putative signal peptide for mitochondrial import (9). However, a systematic analysis of MRPs in HCC prognosis and clinical treatment, particularly the possibility of utilization of serum MRPs as biomarkers, is still intriguing.

Our research classified HCC patients into two subgroups based on the expression of MRPs and investigated the clinical characteristics, immunity and chemotherapy response between them. Further, we focused on MRPL9 and explored the alterations of serum MRPL9 in patients with hepatic disease and healthy volunteers, thus confirming the diagnostic function. In vitro experiments validated the oncogenic roles of MRPL9 in the pathogenesis of HCC.

## Materials and methods

### Patients and sample collection

From November 2020 to July 2021, 49 healthy controls (HCs), 20 benign liver disease patients (BLD) and 78 HCC patients were consecutively enrolled in this study. All serum samples were collected prior to surgery or radiation therapy and rapidly frozen at −80 °C until use, avoiding freezing and thawing cycles.

The diagnosis of HCC was verified by histological testing of the resected material from patients. Clinicopathological parameters of the subjects in this research, including age, gender, CNLC (China liver cancer staging) stage, distant tumor metastasis and tumor size were collected. The institutional ethics committee for human studies at the Second hospital affiliated with Chongqing Medical University granted this study. Informed consent was acquired from all volunteers following the related rules. Patient features were concluded in Table 1.

**Table 1:** The factors of enrolled patients.

Parameters	HC	BLD	HCC
Number of subjects	49	20	78
Age (range)	20–82	28–84	29–85
Gender (male : female)	39 : 10	11 : 9	66 : 12
MRPL9 (pg/ml)	652 (395.8, 857)	955.8 (572.6, 1391)	2,401 (1623, 4404)
AFP (ug/l)	8.64 (5.235, 13.07)	7.71 (3.49, 11.19)	35.15 (6.19, 126.8)
Ferritin (ng/ml)	138.5 (87.15, 177.3)	126.5 (80.48, 168.3)	204.2 (146.8, 365.3)

### Detection of Serum samples

Serum was isolated from the specimens and stocked at −80 °C after centrifuging at 3,000 rpm for 10 min. ELISA Kit (Mlbio, Shanghai, China) was measured following the manufacturer's instructions. The automatic chemiluminescence immunoassay system LIAISON XL (Diasorin, Italy) was applied to detect AFP and Ferritin levels in serum samples.

### Bioinformatic analyses

Clinical data and expression profiles were downloaded from The Cancer Genome Atlas (TCGA) dataset (https://portal.gdc.cancer.gov/) (10) and the International Cancer Genome Consortium (ICGC) (https://dcc.icgc.org/releases/current/Projects). We performed consistency analysis following the expression of MRPs. Log-rank test was conducted to compare survival variance and time-dependent receiver operating characteristic (ROC) curves were performed to compare the predictive accuracy of MRPL9 for the prognosis of HCC patients. Nomogram and decision curve analyses (DCA) validated the prognostic effects. Univariate and multivariate cox regression analysis was applied to access the prognostic effect of risk scores. The half-maximal inhibitory concentrations (IC50) were downloaded from the Genomics of Drug Sensitivity in Cancer (GDSC, https://www.cancerrxgene.org/) (11). The immunophenoscore (IPS) and the tumor immune dysfunction and exclusion (TIDE) algorithm were applied to assess the immunotherapeutic response (12). OCLR algorithm predicted the stemness (13). Gene Ontology (GO) and Kyoto Encyclopedia of Genes and Genomes (KEGG) pathway enrichment analysis were analyzed by the differentially expressed genes (DEGs) in HCC patients. Threshold: fold-change (FC) = 3, *p* < 0.05. Gene Set Enrichment Analysis (GSEA) was conducted to investigate the potential mechanism and biological functions between two clusters. R packages were performed using R software version v4.0.3, including “rms”, “ggrisk”, “survival”, “survminer”, “timeROC”, “pRRophetic”, “clusterprofiler”, “ggplot2”, “immunedeconv”, “ConsensusClusterPlus” and “ggDCA”.

### Cell culture

The liver tumor line cells Huh7 and HepG2 were grown in Dulbecco's modified Eagle's medium (Hyclone, Uinted States) containing 10% FBS (Lonsera, Uruguay) supplemented with 1% penicillin/-streptomycin at 37 °C in a humidified incubator containing 5% CO2. The overexpressed plasmid (EX-V0670-M02-MRPL9) and control plasmid (EX-V0670-M02-Control) were purchased from Labcell biotechnology (Chongqing, China). And they were transfected into Huh7 and HepG2 cells using H4000 (Engreen, China) based on the standard instruction.

### CCK8

Cell proliferation was detected by the cell counting kit-8 (Dojindo Laboratories, Kumamoto, Japan) according to the manufacturer's instructions. In brief, 1,000 cells transfected with MRPL9 overexpressed plasmid and control plasmid were plated into 96-well plates and cultured until the indicated time. Then 10 µl of CCK-8 agent was added to every well and incubated for 1.5 h. The absorbance at 450 nm was detected to calculate the number of viable cells.

### Flow cytometry

Cell cycles were measured by CytoFLEX flow cytometer. Cells were harvested after different treatments and then washed twice with ice-cold PBS, dyed with propidium iodide (PI) followed by flow cytometry analysis, as previously described (14).

### Transwell

The MRPL9-overexpressed cells and control cells were resuspended in an FBS-free medium and seeded in the top chamber of transwell plates at a density of 5 × 10^4^ cells/well, 500 µl of DMEM containing 10%FBS was added in the bottom chamber. After incubating for 24 h at 37 °C with 5% CO_2_, the cells were fixed with 4% formaldehyde and stained with crystal violet. As for the invasion assay, the chamber was coated with Matrigel (40 µl/well, 1 : 5 dilution). The cells were counted by 3 random fields of every well.

### Western blot analysis

Western blotting was performed as described previously (14). The following primary antibodies were used: MRPL9 (15342-1-AP, 1:1000, Proteintech, China), β-Tubulin (10068-1-AP, 1 : 1,000, Proteintech, China), E-cadherin (24E10, 1:800, CST, US), β-catenin (sc-7199, 1:800, Sants Cruz Biotechnology, US), N-cadherin (22018-1-AP, 1:1000, Proteintech, China). The protein signals were visualized using ECL ((Millipore, Massachusetts, Uinted States) and the signal strength was analyzed using Image Lab V6.0 software.

### Statistical analysis

All statistical analyses were conducted using GraphPad Prism software7.0 (GraphPad Software, CA, Uinted States) and the SPSS program (version 23.0). Median values with interquartile ranges were used to describe serum protein concentrations. The Mann–Whitney test was used to compare two groups and the Kruskal–Wallis test to compare serum markers in more than two groups. The chi-square test examined categorical data. ROC analysis was performed to evaluate the diagnostic role. *p*-value < 0.05 was considered as statistical significance. *p* <0.05 (*); *p* < 0.01(**); *p* < 0.001(***).

## Results

### Transcriptional variation of MRPs in HCC

**Figure 1:**
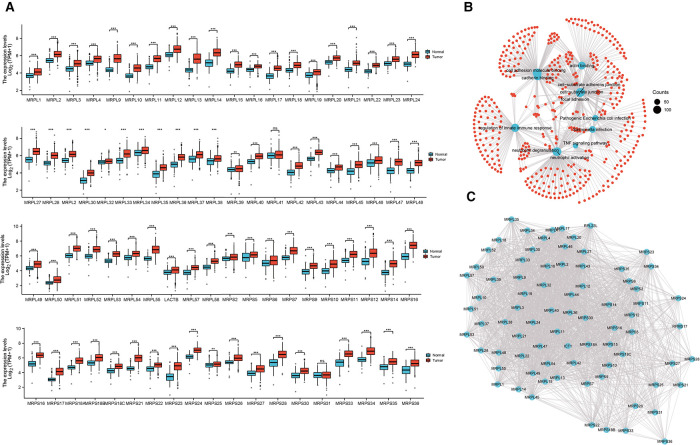
Expression and molecular interaction of MRPs. (**A**) Expression of MRPs in HCC and normal tissues combined with TCGA and GTEx data. (**B**) Enrichment analysis of MRPs. (**C**) PPI analysis of MRPs.

The expression of 79 MRPs in HCC was detected according to the TCGA and GTEx profiles. The overall survival (OS) prognosis roles of MRPs in HCC patients were presented in Supplementary Table S1 and high levels of 37 MRPs indicated poor outcomes. Our results signified that 78 MRPs were remarkably augmented in tumor tissues than in normal individuals (Figure 1A). Functional enrichment profiling suggested that MRPs mainly got involved in the cellular adheren junction and innate immune response (Figure 1B). Protein-protein interaction (PPI) plot revealed the interactivity of MRPs (Figure 1C).

### Generation of MRPs related molecular subtypes in HCC

**Figure 2:**
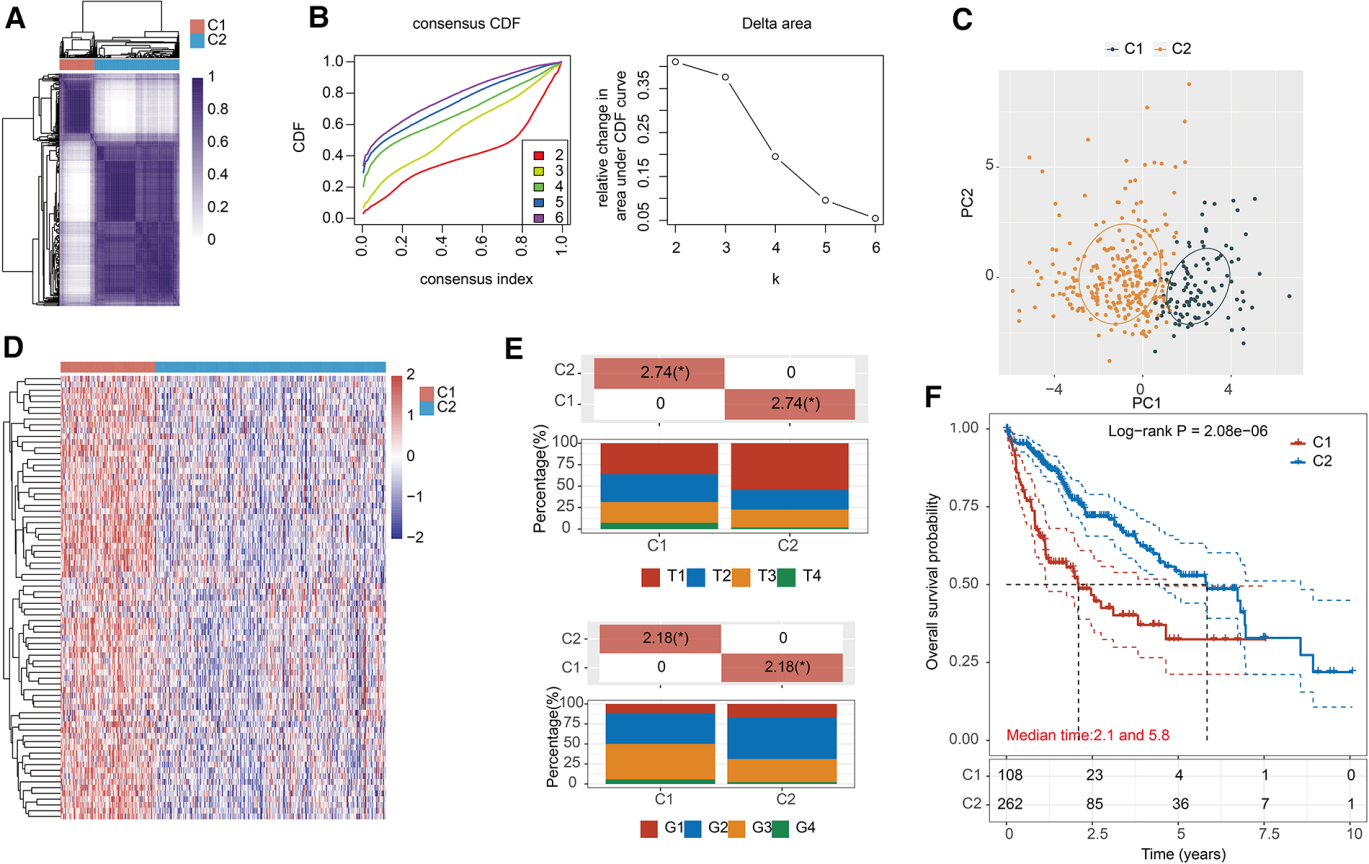
Identification of MRPs associated subtypes in HCC. (**A**) The consensus matrix analysis classified two clusters, cluster 1 (*n* = 108), cluster 2 (*n* = 263). (**B**) Consensus clustering cumulative distribution function (CDF) and relevant variations in the area under the CDF curve. (**C**) PCA revealed the distribution of patients in two classifiers. (**D**) The heatmap signified the levels of MRPs in two subtypes. (**E**) The bar chart presented the clinicopathological differences in the two clusters. (**F**) KM curve verified the OS difference in MRPs clusters.

371 HCC patients were enrolled and we performed a consensus clustering analysis to sort them into two clusters based on the expression of MRPs (Figures 2A,B, Supplementary Table S2). Principal component analysis (PCA) demonstrated the superior intergroup distribution (Figure 2C). Heatmap showed that the genomic levels of MRPs were higher in cluster 1 than in cluster 2 (Figure 2D). The bar chart displayed that the T stage and grade were significantly different between the two subtypes (Figure 2E). Additionally, as determined in Figure 2F, the KM curve indicated that patients in cluster 1 had a poorer prognosis than those in cluster 2.

### Functional enrichment analysis

**Figure 3:**
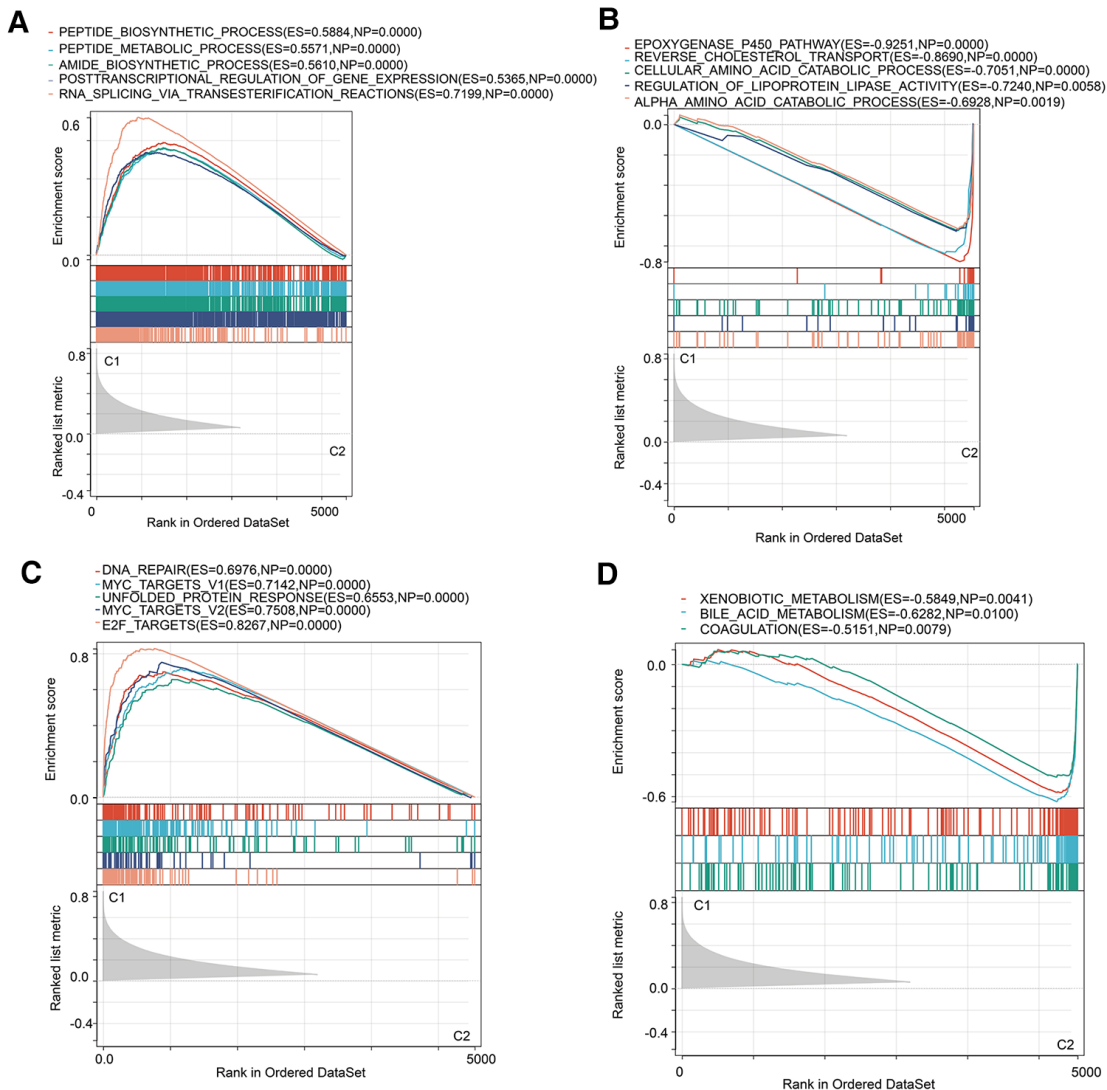
GSEA analysis in two MRPs clusters. (**A,B**) Hallmark pathways in two MRPs molecular subtypes. (**C,D**) Biological processes analysis in two MRPs clusters.

Subsequently, we investigated the underlying biological activity and pathways in two subtypes. GSEA of biological processes implied that cluster 1 was majorly enriched in peptide and amide biosynthetic processes, and the regulation of gene expression (Figure 3A). Catabolic processes were involved in cluster 2 (Figure 3B). In the following pathways analysis, we derived DNA repair, E2F targets and MYC targets (Figure 3C). As for cluster 2, we mainly observed metabolism-associated pathways (Figure 3D). Moreover, we performed KEGG and GO analyses on the differentially expressed genes in two molecular subtypes and found cell cycle was the key term in upregulated genes (Supplementary Figure S1A). Downregulated genes mainly participated in the metabolism-related signal transduction (Supplementary Figure S1B). GO analysis of upregulated genes was primarily enriched in organelle fission and nuclear division (Supplementary Figure S1C), and downregulated molecules were concerned with multiple metabolism processes (Supplementary Figure S1D).

### Drug susceptibility analysis

**Figure 4:**
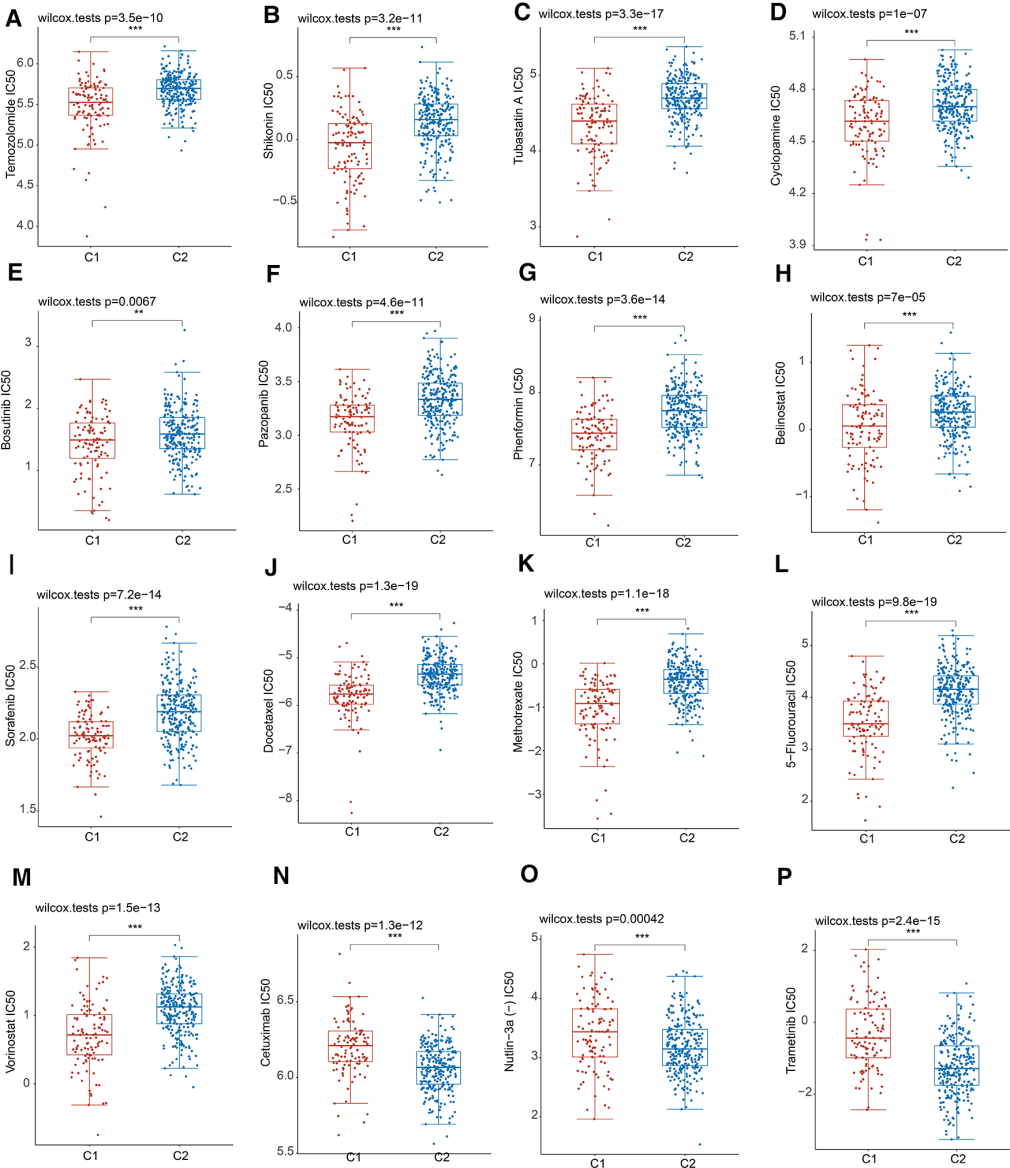
Drug sensitivity analysis in two MRPs groups. (**A**) Temozolomide. (**B**) Shikonin. (**C**) Tubastatin A. (**D**) Cyclopamine. (**E**) Bosutinib. (**F**) Pazopanib. (**G**) Phenformin. (**H**) Belinostat. (**I**) Sorafenib. (**J**) Docetaxel. (**K**) Methotrexate. (**L**) 5-Fluorouracil. (**M**) Vorinostat. (**N**) Cetuximab. (**O**) Nutlin-3a (−). (**P**) Trametinib.

Resistance to chemotherapy is a major obstacle to effective cancer treatment (15). Considering the diverse clinical features and prognosis, we further calculated the IC50 values of 16 common anticancer drug agents in two MRPs clusters by applying the pRRophetic algorithm. Among the 16 drugs, 13 of them might be more sensitive for patients in cluster 1, including Temozolomide, Shikonin, Tubastatin A, Cyclopamine, Bosutinib, Pazopanib, Phenformin, Belinostat, Sorafenib, Docetaxel, Methotrexate, 5-Fluorouracil, Vorinostat (Figures 4A–M). As regards to the patients in cluster 2, Cetuximab, Nutlin-3a (−) and Trametinib might be more efficient for the treatment (Figures 4N–P).

### Prediction of immunity and immunological therapeutic response

**Figure 5:**
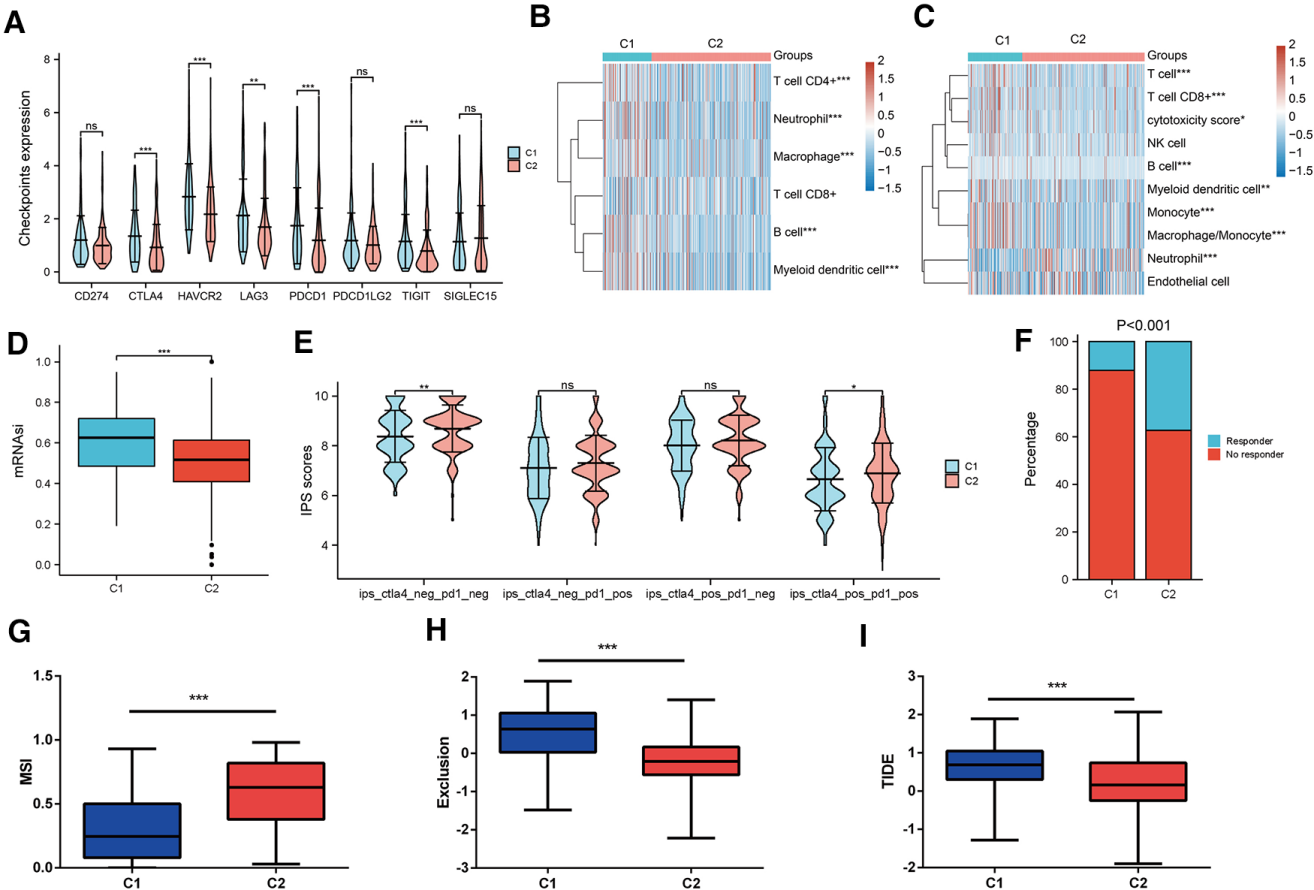
Prediction of TME and ICB response in two MRPs related subtypes. (**A**) Differential expression of immune checkpoints in two groups. (**B**) TIMER and (**C**) MCPCOUNTER algorithms estimated the levels of immune cell infiltration. (**D**) OCLR algorithm calculated the stemness scores in two clusters. (**E**) IPS predicted the anti-PD1 and anti-CTLA4 therapy response in two molecular subtypes. (**F**) TIDE algorithm assessed the percentage of responders. (**G**) MSI, (**H**) Exclusion scores, (**I**) TIDE scores across two subtypes.

Then, we estimated the characteristics of the tumor microenvironment between the two clusters. As shown in Figure 5A, the majority of immune checkpoints were notably elevated in cluster 1. TIMER and MCPCOUNTER algorithms uncovered that the abundance of B cells, CD8+ T cells, Macrophage and Myeloid dendritic cells were enriched in cluster 1 (Figures 5B,C). Simultaneously, the stemness scores were significantly increased in cluster 1 (Figure 5D). Besides, immunophenoscore (IPS) confirmed that patients in cluster2 might benefit more from anti-PD1 and anti-CTLA4 therapy (Figures 5E,F). The TIDE algorithm validated that patients in subtype2 were conferred higher microsatellite instability and exclusion scores (Figures 5G,H), while having lower TIDE scores (Figure 5I). Our findings suggested that cluster 2 may have a higher likelihood of a response to ICB response, thereby achieving a better clinical outcome.

### Construction and validation of prognostic model

**Figure 6:**
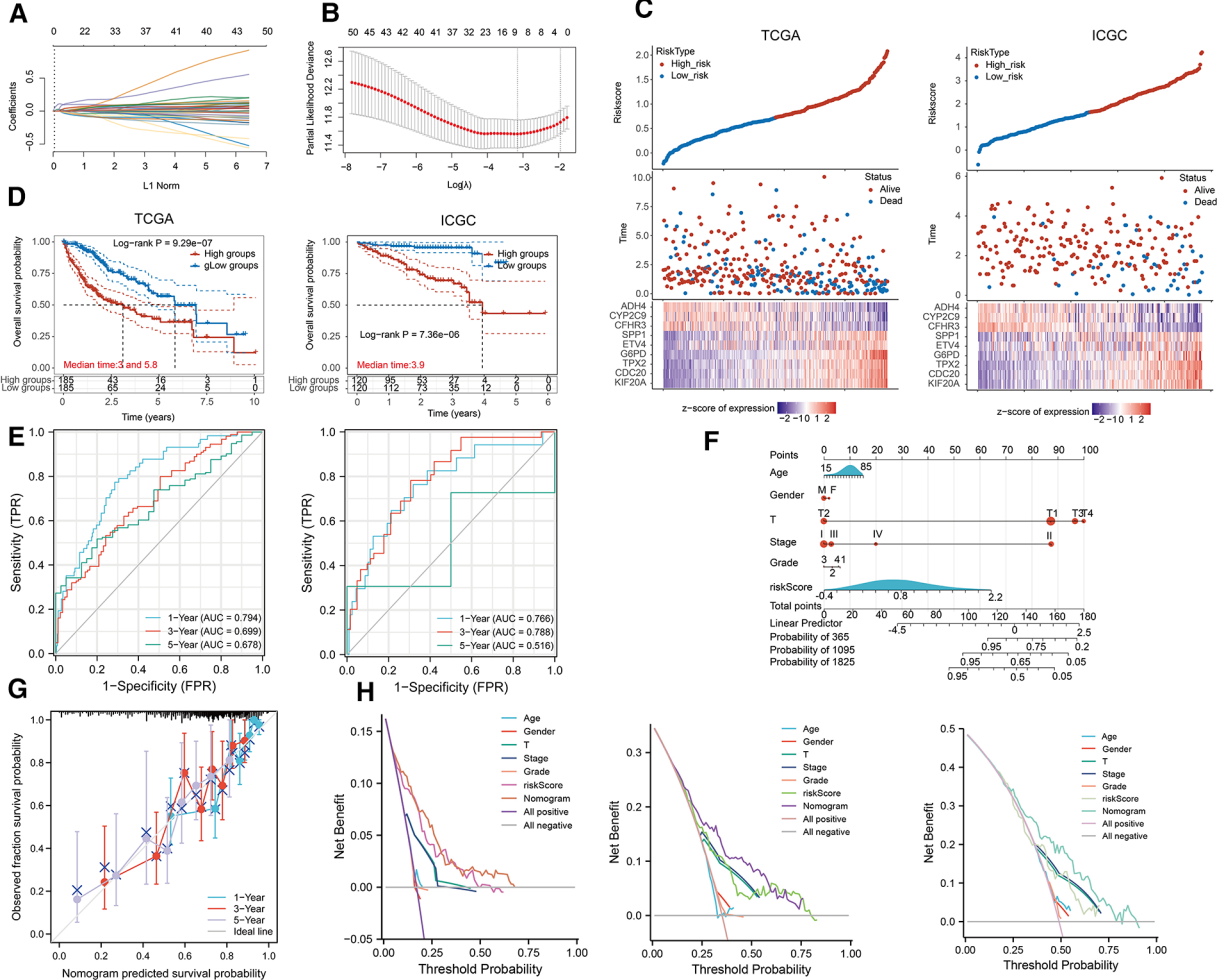
Development of prognostic model based on DEGs. (**A,B**) LASSO Cox regression model. (**C**) Heatmap illustrated the distribution of HCC patients according to their status in TCGA and ICGC profiles. (**D**) KM curves for the different groups of risk scores. (**E**) ROC curves for the short and long terms prediction of prognosis. (**F**) Construction of a nomogram by integrating clinical factors and risk scores. (**G**) The calibration plots for the calculation of 1-, 3- and 5-year prognosis. (**H**) DCA validated the performance of the nomogram in evaluating 1-, 3- and 5-year OS.

**Figure 7:**
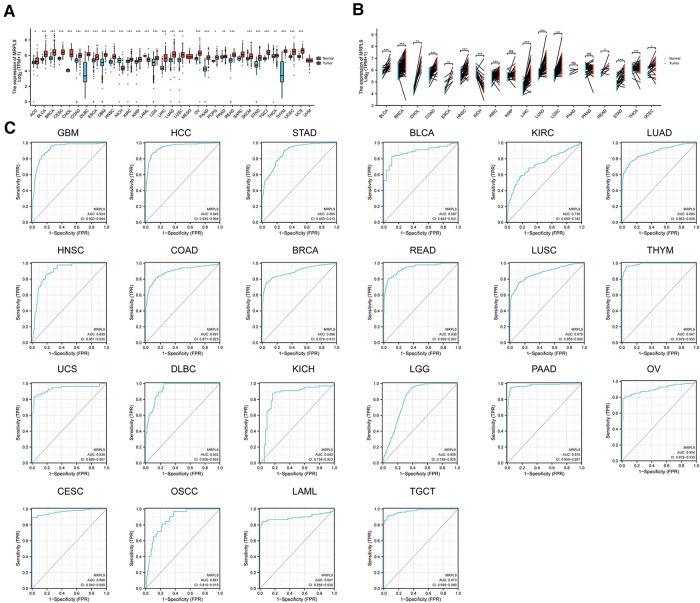
Expression and diagnostic roles of MRPL9 in the majority of tumors. (**A**) Transcriptional levels of MRPL9 in pan-cancer based on TCGA and GTEx datasets. (**B**) Expression of MRPL9 in tumor tissues and paired normal tissues based on TCGA files. (**C**) ROC plots illustrated the potential of MRPL9 in oncology diagnosis.

Afterward, we implemented LASSO regression analysis to develop a prognostic signature following DEGs (Figures 6A,B, Supplementary Table S3). The model apparently differentiated the status of HCC patients in TCGA and we validated it in ICGC databases (Figure 6C). KM plots revealed that patients with high risk had an adverse OS than the low-risk group (Figure 6D). ROC plots exerted satisfactory effects in predicting prognosis for short and long terms (Figure 6E). Univariate and multivariate Cox analysis demonstrated our risk score presented as an independent predictive factor (Supplementary Table S4). Additionally, we incorporated clinical parameters to construct a nomogram for the prediction of 1-, 3- and 5-year prognosis (Figure 6F). The calibration curve approximated the desired curve (Figure 7G) and DCA demonstrated a greater advantage in the prediction of the prognosis in 1-, 3- and 5-year than other clinical factors (Figure 6H).

### Expression and diagnostic roles of MRPL9 in pan-cancer

Given the aberrant expression and prognostic roles of MRPL9, we further validated the promising clinical values and the carcinogenic effects in HCC. The expression of MRPL9 in multiple tumors based on TCGA and GTEx datasets were explored and the results indicated that MRPL9 was highly expressed in adrenocortical carcinoma (ACC), bladder urothelial carcinoma (BLCA), breast invasive carcinoma (BRCA), cervical squamous cell carcinoma and endocervical adenocarcinoma (CESC), cholangio carcinoma (CHOL), colon adenocarcinoma (COAD), esophageal carcinoma (ESCA), lymphoid neoplasm diffuse large B-cell lymphoma (DLBC), esophageal carcinoma (ESCA), glioblastoma multiforme (GBM), head and neck squamous cell carcinoma (HNSC), kidney renal clear cell carcinoma (KIRC), kidney renal papillary cell carcinoma (KIRP), brain lower grade glioma (LGG), lung adenocarcinoma (LUAD), lung squamous cell carcinoma (LUSC), ovarian serous cystadenocarcinoma (OV), prostate adenocarcinoma (PRAD), pancreatic adenocarcinoma (PAAD), pheochromocytoma and paraganglioma (PCPG), skin cutaneous melanoma (SKCM), rectum adenocarcinoma (READ), testicular germ cell tumors (TGCT), stomach adenocarcinoma (STAD), thyroid carcinoma (THCA), thymoma (THYM), uterine corpus endometrial carcinoma (UCEC) and uterine carcinosarcoma (UCS); while was decreased in kidney chromophobe (KICH) and acute myeloid leukemia (LAML) compared with normal tissues (Figure 7A). Similar results were observed when compared with corresponding adjacent normal tissues (Figure 7B). Besides, we found that MRPL9 could exhibit excellent diagnostic function in diverse tumors (Figure 7C).

### The diagnostic potential of Serum MRPL9 for HCC patients

**Figure 8:**
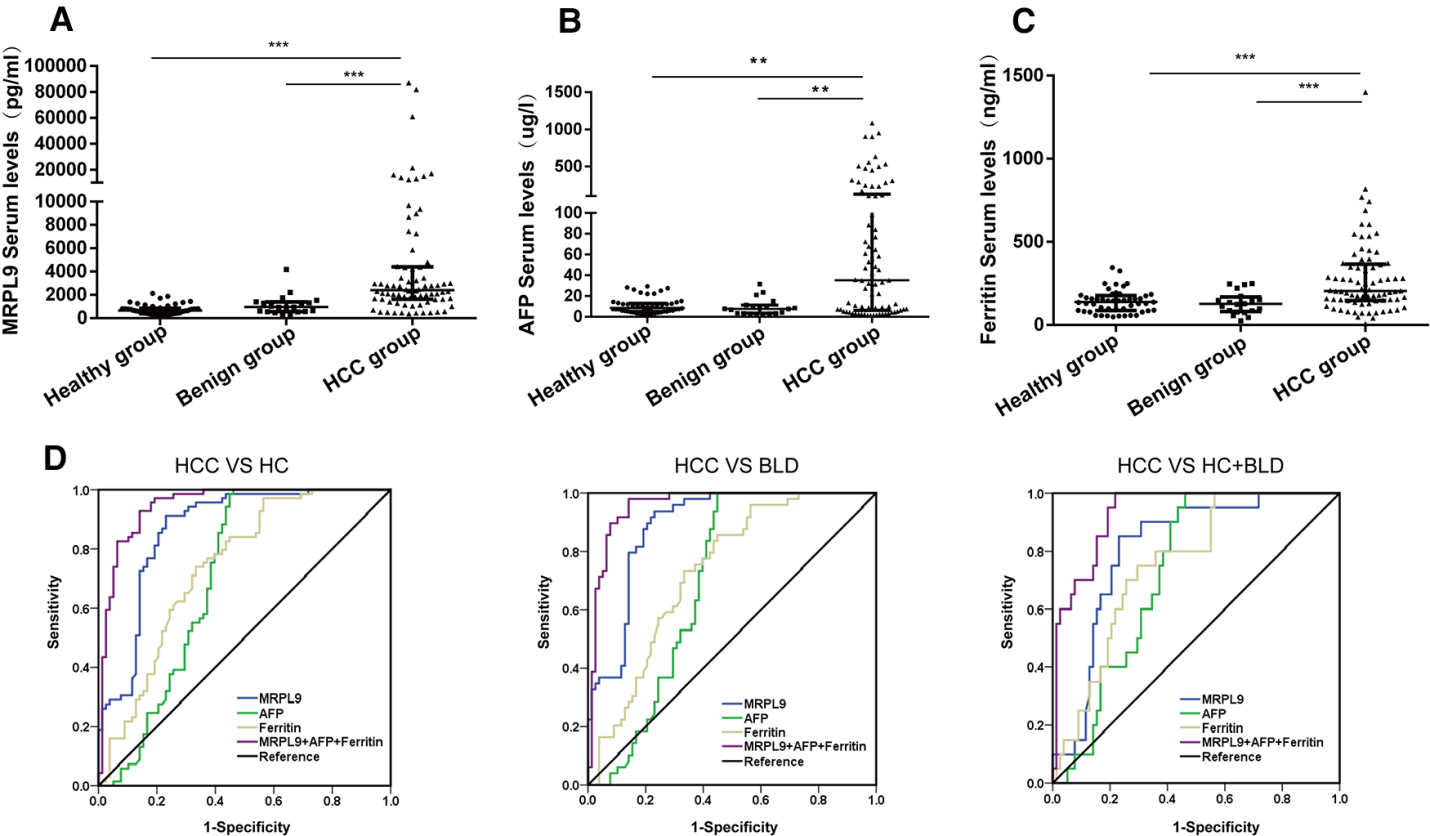
Serum MRPL9, AFP and ferritin levels in the healthy, benign liver disease, HCC groups and ROC curves. (**A–C**) MRPL9, AFP and Ferritin serum levels in healthy, benign liver disease and HCC groups, respectively. (**D**) ROC curves were constructed to predict the diagnostic accuracy of MRPL9 for distinguishing HCC patients from non-HCC subjects, healthy group and benign liver disease group, respectively.

Earlier analysis implied the potential of MRPL9 in diagnosis for HCC patients, we further validated the serum diagnostic values. AFP and Ferritin were widely used serum biomarkers to detect HCC worldwide. We analyzed the serum MRPL9, AFP and Ferritin levels in HCC, benign liver disease and healthy groups. As shown in Figures 8A–C, serum MRPL9, AFP and Ferritin levels were significantly higher in HCC patients than in benign liver disease patients and healthy individuals (*p* < 0.01). ROC analysis was conducted to test the diagnostic accuracy of serum MRPL9. The area under curve (AUC) value was calculated to evaluate the diagnostic efficacy (16). The curve analysis indicated that MRPL9 had a high AUC value (0.867) to distinguish HCC patients from non-cancerous individuals (Figure 8D). When the cut-off value was 1581.3 pg/ml, the sensitivity and specificity were 76.9% and 91.3%, respectively. In contrast, both AFP and Ferritin exhibited low AUC values (0.705 and 0.74, respectively) and low sensitivity (53.8% and 43.6%, respectively), which suggested that MRPL9 was superior to AFP and Ferritin in distinguishing HCC from healthy and benign groups. Most importantly, the combination of these three markers resulted in a considerable increase in AUC value (0.948), with high sensitivity and specificity of 85.9% and 92.8%, respectively. Similarly, MRPL9 also had the best diagnostic potency in distinguishing HCC from the healthy (AUC = 0.89) and benign liver disease groups (AUC = 0.812) than AFP (AUC = 0.695, 0.729, respectively) and Ferritin (AUC = 0.734, 0.757 respectively; Supplementary Tables S5–S7).

### The association between Serum MRPL9 level and the clinicopathological factors of HCC patients

Given the increased level of serum MRPL9 in HCC patients, we further determined the correlation between MRPL9 and different clinicopathological factors, including gender, age, tumor size, CNLC stage and HCC diagnostic markers including AFP, Ferritin. The serum MRPL9 was associated with tumor size and distant tumor metastasis. And there was no statistical difference in other clinical parameters. These results were shown in Table 2.

**Table 2:** Correlations of serum MRPL9 level and different clinical pathological parameters of HCC.

Parameters	Groups	*n*	MRPL9 (pg/ml)	*p*-value
Gender	Male	66	2,401 (1,703, 4,259)	0.9415
	Female	12	2,038 (1,109, 15,028)	
Age	≤50	31	2,312 (1,142, 4,231)	0.338
	>50	47	2,746 (1,692, 4,447)	
Tumor size	<3 cm	36	2,401 (1,684, 4,433)	0.5715
	≥3 cm	42	2,301 (1,314, 4,725)	
Stage	I + II	38	2,012 (940.6, 3,303)	0.0316
	III + IV	40	2,765 (2,022, 4,710)	
Metastasis	Yes	53	2,785 (1,794, 5,333)	0.0201
	No	25	2,041 (623.7, 2,725)	
AFP	≤200	60	2,323 (1,657, 4,316)	0.4186
	>200	18	2,776 (1,732, 6,647)	
Ferritin	≤400	63	2,319 (1,403, 4,390)	0.4346
	>400	15	2,784 (1,935, 5,869)	

### Overexpressed MRPL9 promotes liver cancer cell proliferation, migration, and invasion *in vitro*

**Figure 9:**
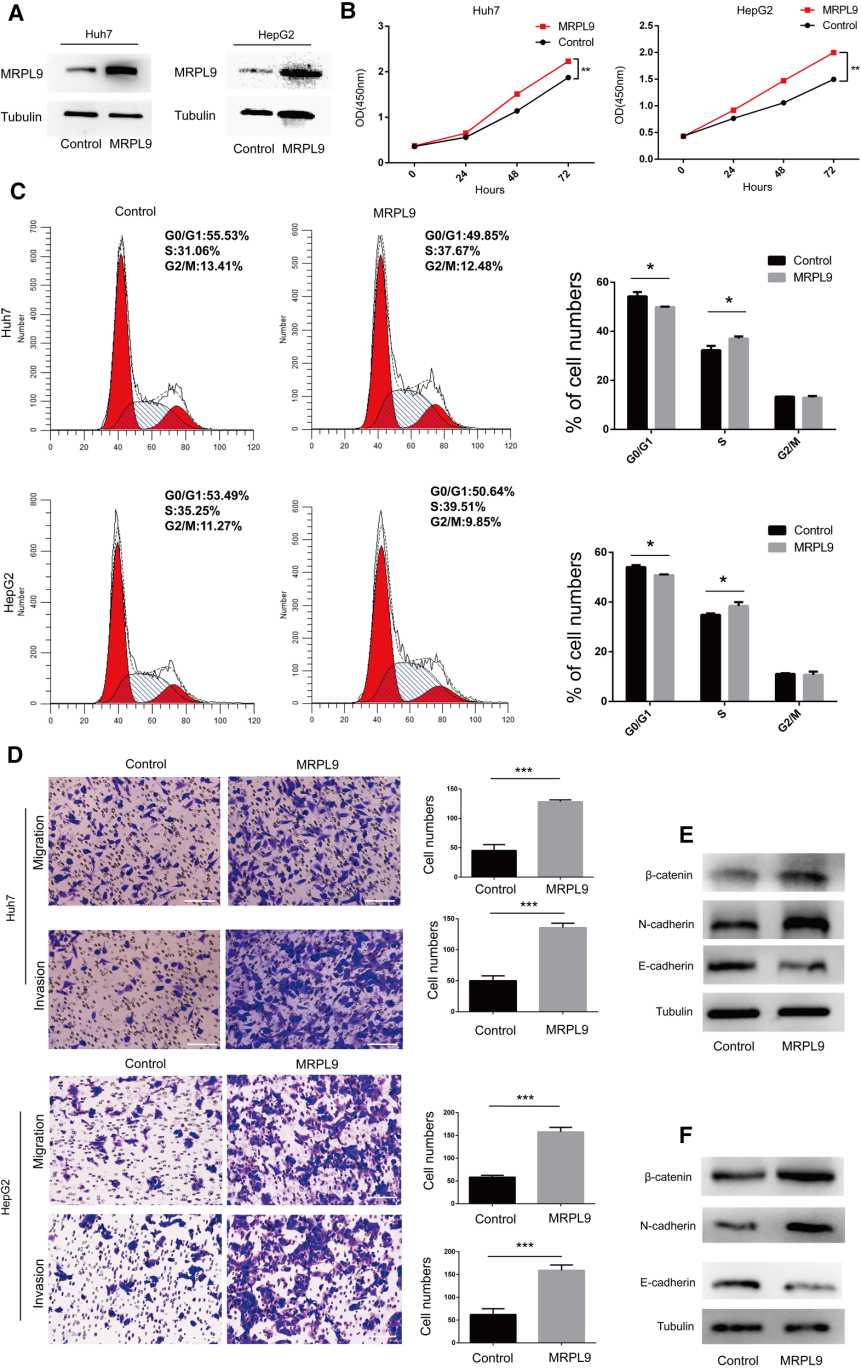
Overexpressed MRPL9 promotes liver cancer cell proliferation, migration, and invasion progression. (**A**) Overexpressed MRPL9 was verified by western blot in Huh7 and HepG2 cells. (**B**) MRPL9 promotes the ability of proliferation in Huh7 and HepG2 cells. (**C**) Flow cytometry indicated that overexpressed MRPL9 promoted the transition of the cell cycle of Huh7 and HepG2 cells. (**D**) Overexpressed MRPL9 promoted the migration and invasion of Huh7 and HepG2 cells. Scale bar = 100 µm. (**E,F**) Western blot revealed that MRPL9 could promote the process of EMT in Huh7 and HepG2 cells, respectively.

Next, the MRPL9 overexpressed and control plasmids were transfected into Huh7 and HepG2 cells, and the higher expression level of MRPL9 was identified by western blot (Figure 9A). Then, the CCK8 assay showed an enhanced proliferation rate in the overexpressed group compared to the control group (*p* < 0.01, Figure 9B). Upregulated MRPL9 promoted the transition of the G1/S phase (Figure 9C). Transwell assay showed that overexpressed MRPL9 promoted the ability of migration and invasion in Huh7 and HepG2 cells (*p* < 0.05, Figure 9D). Furthermore, western blot demonstrated overexpressed MRPL9 could promote the epithelial-mesenchymal transition (EMT) process in Huh7 and HepG2 cells (Figures 9E,F).

## Discussion

Based on recent studies, the MRPs and their coding genes have been found to be highly correlated with many diseases. Multiple MRPs may be a promising prognostic factor for the diagnosis of disease (3, 17–20). However, there is still a lack of extensive and in-depth research on the role of MRPs in tumors, especially in HCC.

In our study, we systematically probed the molecular expression and interaction patterns. Our findings discovered that mostly MRPs were prominently augmented in tumor tissues with poor OS. Because of phenotypic variability and heterogeneity, there is a wide range of limitations in the management of HCC (21, 22). Integrative multi-omics analyses provided actionable targets for drug application as well as biomarkers for response prediction (23). Differentiation of different subtypes in HCC is a precondition for the delivery of individualized medicine. Consequently, we used a consensus clustering algorithm to classify HCC patients into two types with distinct molecular features and clinical characteristics according to the integrated levels of MRPs. Intriguingly, GSEA showed that patients in two subtypes were conferred different biological functions and accompanied by diverse cancer-related pathways. As HCC is highly heterogeneous clinically, the revolution of chemotherapy needs to be recognized in individualized treatment. Taking into account the distinct discrepancy in the two subtypes, we further calculated the IC50 values of 16 common anticancer agents. Notably, they all showed significant differences with respect to the two MRPs-related molecular subtypes, including sorafenib, the first systemic agent approved for the treatment of advanced-stage HCC. Accordingly, MRPs-related subtypes have potential for individualized chemotherapy and may benefit HCC patients to a certain degree.

Currently, accumulative evidence suggested that the interaction of tumor cells with variable TME elements may contribute to the shedding of cancer and ultimately result in the proliferation, recurrence and metastasis of cancer (24, 25). Immune checkpoint blockade (ICB) has transformed the landscape of cancer management. Nevertheless, only a small proportion of patients have a primary response (26–28). Thus, we evaluated the abundance of two MRPs-associated subtypes with TIMER and MCPCOUNTER algorithms. Our results implied that patients with high MRPs levels had more possibilities of immunological escape and exclusion scores. OCLR algorithm identified that patients in cluster 1 had higher stemness scores indicating that they may have a higher risk of metastasis and treatment resistance (29, 30). IPS and TIDE scores are promising biomarkers in ICB for many patients with tumors (31, 32). Our study demonstrated that patients with low MRPs had lower stemness scores and immunological rejection levels, which might benefit more from immunotherapy, thus with better outcomes.

One of the most common constituents of cancer, HCC, is difficult to detect at an early stage (1). AFP and ferritin have been widely used in HCC detection. However, AFP and ferritin levels were not significantly increased in HCC (33, 34). Therefore, it is imperative to search for a novel type of serum diagnosis for the early detection of HCC. Bioinformatics files illustrated that MRPL9 levels have increased in most cancers, including HCC. Uniformly, the serum concentration of MRPL9 in HCC patients was significantly enhanced compared with early liver diseases and healthy individuals. The ROC curve analysis showed that MRPL9 was superior to the other two normal serum biomarkers (AUC = 0.867) in differentiating HCC from non-cancer subjects. In view of the low sensitivity or specificity of a single marker, the combination of tumor markers has been used to increase the accuracy of diagnosis (35, 36). In this study, we demonstrated that MRPL9, AFP, and Ferritin were used to increase the diagnostic efficacy, which resulted in the highest diagnostic accuracy (AUC = 0.948) and sensitivity (85.9%) while retaining a high degree of specificity (92.8%).

To elucidate the role of MRPL9 in HCC, we transfected overexpressed MRPL9 plasmid into Huh7 and HepG2 cells and found that upregulated MRPL9 could significantly promote tumor proliferation, metastasis and interfere cell cycle by promoting the transition of G1/S phase. Cell cycle disruption could lead to infinite proliferation, which might play a critical role in liver cancer and a promising therapeutic target (37, 38), suggesting that MRPL9 may function a critical role in the tumorigenesis of HCC. Moreover, MRPL9 could accelerate EMT progression, which is very important in HCC's early stage of metastasis (39). Surgical treatment such as resection or liver transplantation occupied influential roles in HCC despite the increased recurrence rates (40, 41). Identification of applicable indicators for recurrence determination was of great implications (42). In silico analysis and *in vitro* experiments confirmed that MRPs were implicated in immune response modulation and tumorigenesis. Considering the prominent contribution to malignant phenotypes and elevated levels of almost all MRPs, we reasonably speculated that MRPs might be also exploited for monitoring the relapse possibility and pave the way for surgical management.

Inevitably, there are certain limitations to our study. In vivo experiments are required to confirm the functions and mechanisms of MRPL9 in HCC. Although there is notable differentiation for ICB and chemotherapy between two MRPs molecular subtypes based on machine learning, it required validation from the clinical cohort. And more clinical specimens and subgroups will be involved to verify the prognosis and diagnosis.

## Conclusion

In conclusion, we identified two MRPs-related molecular subtypes in HCC which had distinct clinical characteristics, biological functions, immunity and prognosis. Patients in two clusters were conferred diverse responses to immunotherapy and chemotherapy. Among them, MRPL9 demonstrated a more accurate diagnosis of HCC than that of AFP and Ferritin, and increased expression of MRPL9 may contribute to HCC progression. Our molecular subtypes could offer a new perspective in the field of precision medicine, and MRPL9 could be used as a substitute for the diagnosis of HCC.

## Data Availability

The datasets presented in this study can be found in online repositories. The names of the repository/repositories and accession number(s) can be found in the article/**Supplementary Material**.
