# Supplementary material for: Classification of the mitochondrial ribosomal protein-associated molecular subtypes and identified a serological diagnostic biomarker in hepatocellular carcinoma
Source: Front Surg. 2023 Jan 6;9:1062659. doi: 10.3389/fsurg.2022.1062659 (PMC9853988; doi:10.3389/fsurg.2022.1062659)
Supplement: Supplementary file 1 [file Datasheet1.zip › code_KM.docx]

load('XXX.RData')

cli.eac=read.csv('PMC6066282-TCGA-CDR-clinical.txt',stringsAsFactors = F,row.names = 1,check.names = F,sep = '\t')

comsample=intersect(paste0(rownames(cli.eac),'-01'),colnames(dt))

data.ana=log2(dt[,comsample]+1)

data.cli=cli.eac[substr(comsample,1,12),]

########################################################################################

library(glmnet)

times=data.cli$OS.time

status=data.cli$OS

genes=c('KIF18A','KIF15','KIF14','KIF4A','KIF2C')####################输入基因列表补充到这里

set.seed(250)

fit1=glmnet(as.matrix(t(data.ana[genes,]))

#,factor(samps)

,cbind(time=times,status=status)

,family="cox"

#,family="binomial"

#,type.measure="deviance"

,nlambda=100

, alpha=1)

set.seed(250)

cv.fit<-cv.glmnet(as.matrix(t(data.ana[genes,]))

#,factor(samps)

,cbind(time=times,status=status)

,family="cox"

#,family="binomial"

#,type.measure="deviance"

,nlambda=100

, alpha=1)

sig.coef=coefficients(cv.fit,s=cv.fit$lambda.min)[which(coefficients(cv.fit,s=cv.fit$lambda.min)[,1]!=0),1]

pdf('Figure1.pdf',width = 8,height = 6)

par(mfrow=c(1,2))

plot(fit1, label = TRUE)

plot(cv.fit)

dev.off()

###

lan=sig.coef

genes=names(sig.coef)

risk=as.numeric(lan%*%as.matrix(data.ana[genes,]))

library(survival)

library(survminer)

library(pheatmap)

library(survcomp)

library(ggplot2)

coxRun=function(dat){

library(survival)

colnames(dat)=c('time','status','AS')

dat=dat[which(!is.na(dat[,1])&!is.na(dat[,3])&!is.na(dat[,2])),]

#print(nrow(dat))

if(nrow(dat)<10){

print(paste0('Sample Num is small:',nrow(dat)))

return(c(NA,NA,NA,NA))

}

#if(quantile(dat[,3])['25%']==quantile(dat[,3])['50%']) return(c(NA,NA,NA,NA))

fmla <- as.formula("Surv(time, status) ~AS")

if(table(dat[,2])[1]>1&table(dat[,2])[2]>1){

cox <- coxph(fmla, data = dat)

re=c(summary(cox)[[7]][5],summary(cox)[[7]][2],summary(cox)[[8]][3],summary(cox)[[8]][4])

return(re)

}else{

return(c(NA,NA,NA,NA))

}

}

dat=data.frame(times/365,status,ifelse(risk>median(risk),'High Exp','Low Exp'))

colnames(dat)=c('time','status','group')

fit1 <- survfit( Surv(time, status) ~ group,data = dat )

p1=ggsurvplot(fit1,data = dat,pval = F,conf.int =T,conf.int.style ="step"

,pval.coord=c(1, 0.2)

,surv.median.line='hv'

,palette=c('red','blue')

,risk.table='absolute'

,fontsize=3)

p11=p1$plot+theme_bw()+theme(axis.text.y=element_text(family="serif",face="plain",size = 10)

,axis.text.x=element_blank()

,axis.title.x=element_blank()

,plot.margin=unit(c(0.2, 0.2, 0, 0.1), "inches")

#,axis.title.y=element_blank()

,legend.position=c(1,1), legend.justification=c(1,1)

,legend.background = element_rect(fill = NA, colour = NA)

,panel.grid.major = element_blank()

,panel.grid.minor = element_blank()

,legend.title = element_text(family="serif",face="plain",size = 10)

,legend.text = element_text(family="serif",face="plain",size = 10))

median_labels=c()

for(st in unique(p1$data.survplot$strata)){

st1=p1$data.survplot[which(p1$data.survplot$strata==st),]

x_m=-1

if(min(st1$surv)<0.5){

inds=which(st1$surv==0.5)

if(length(inds)>0){

x_m=st1$time[inds[1]]

}else{

x_m=max(st1$time[st1$surv>=0.5])

}

}

if(x_m>0){

median_labels=c(median_labels,round(x_m,1))

}

}

txt_median=p1$data.survplot[1:length(median_labels),]

txt_median[,5]=rep(0.5,length(median_labels))

txt_median[,1]=median_labels

txt_median$Text=unique(paste0('Median time:',median_labels[1],' and ',median_labels[2]))

p11=p11+geom_text(data=unique(txt_median),aes(0,0,label=Text),family="serif",face="plain"

,color="red",hjust=0, vjust=0,size=4)

gp=sort(unique(dat[,3]),decreasing = T)

vls=1:length(gp)

gvls=vls[match(dat[,3],gp)]

g.cox=coxRun(data.frame(dat[,1],dat[,2],gvls))

add_text=paste0('Log-rank P = ',signif(g.cox[1],digits = 3),'\n','HR(',gp[2],')=',round(g.cox[2],3)

,'\n 95%CI(',round(g.cox[3],3)

,', ',round(g.cox[4],3),')')

text.tb=p1$data.survplot[1,]

text.tb[1,1]=0

text.tb[1,5]=0

text.tb$Text=add_text

p11=p11+geom_text(data=text.tb,aes(round(max(dat$time)*0.55,0),1,label=Text),family="serif",face="plain"

,color="black",hjust=1, vjust=1,size=3.5)

p2=p1$table+theme_bw()+theme(axis.text.y=element_text(family="serif",face="plain",size = 10)

,axis.text.x = element_text(family="serif",face="plain",size = 10)

#,axis.text.x=element_blank()

#,axis.title.x=element_blank()

#,axis.title.y=element_blank()

,plot.margin=unit(c(0, 0.2, 0.2, 0.1), "inches")

,plot.title=element_blank()

,legend.position=c(1,1), legend.justification=c(1,1)

#,legend.background = element_rect(fill = NA, colour = NA)

,legend.title = element_text(family="serif",face="plain",size = 10)

,legend.text = element_text(family="serif",face="plain",size = 10))+xlab('Time (years)')

p2=p2+theme(text=element_text(size=12,family="serif"))

g2=ggpubr::ggarrange(p11,p2, ncol = 1, nrow = 2,heights = c(1,0.3),align = "v")

g2

colsr=ifelse(risk[order(risk)]>=median(risk),"firebrick3","navy")

t1=risk-median(risk)

plot(unlist(t1[order(t1)]),pch=16,ylab='RiskScore',col=colsr,type='o')

times1=times[order(risk)]

status1=status[order(risk)]

plot(unlist(times1/365),ylim=c(0,max(times1/365))

,col=ifelse(status1==1,'firebrick3','navy'),pch=16,ylab='Overall Survival'

,cex=1)

legend("topright",legend = c("Dead","Alive"),col=c("firebrick3","navy"),pch=16,cex=1)

bk=unique(c(seq(-2,2, length=100)))

pheatmap(data.ana[genes,order(t1)],breaks = bk,cluster_cols = F,scale = 'row'

,color = colorRampPalette(c('navy','white','firebrick3'))(100)

,show_colnames = F,cluster_rows = F)
