# Supplementary material for: Classification of the mitochondrial ribosomal protein-associated molecular subtypes and identified a serological diagnostic biomarker in hepatocellular carcinoma
Source: Front Surg. 2023 Jan 6;9:1062659. doi: 10.3389/fsurg.2022.1062659 (PMC9853988; doi:10.3389/fsurg.2022.1062659)
Supplement: Supplementary file 1 [file Datasheet1.zip › DCA.docx]

# source: https://www.mskcc.org/departments/epidemiology-biostatistics/biostatistics/decision-curve-analysis

stdca <- function(data, outcome, ttoutcome, timepoint, predictors, xstart=0.01, xstop=0.99, xby=0.01,

ymin=-0.05, probability=NULL, harm=NULL,graph=TRUE, intervention=FALSE,

interventionper=100, smooth=FALSE,loess.span=0.10,cmprsk=FALSE) {

# LOADING REQUIRED LIBRARIES

require(survival)

require(stats)

#ONLY KEEPING COMPLETE CASES

data=data[complete.cases(data[c(outcome,ttoutcome,predictors)]),c(outcome,ttoutcome,predictors)]

# outcome MUST BE CODED AS 0 AND 1

if ((length(data[!(data[outcome]==0 | data[outcome]==1),outcome])>0) & cmprsk==FALSE) {

stop("outcome must be coded as 0 and 1")

}

# data MUST BE A DATA FRAME

if (class(data)!="data.frame") {

stop("Input data must be class data.frame")

}

# xstart IS BETWEEN 0 AND 1

if (xstart<0 | xstart>1) {

stop("xstart must lie between 0 and 1")

}

# xstop IS BETWEEN 0 AND 1

if (xstop<0 | xstop>1) {

stop("xstop must lie between 0 and 1")

}

# xby IS BETWEEN 0 AND 1

if (xby<=0 | xby>=1) {

stop("xby must lie between 0 and 1")

}

# xstart IS BEFORE xstop

if (xstart>=xstop) {

stop("xstop must be larger than xstart")

}

#STORING THE NUMBER OF PREDICTORS SPECIFIED

pred.n=length(predictors)

#IF probability SPECIFIED ENSURING THAT EACH PREDICTOR IS INDICATED AS A T OR F

if (length(probability)>0 & pred.n!=length(probability)) {

stop("Number of probabilities specified must be the same as the number of predictors being checked.")

}

#IF harm SPECIFIED ENSURING THAT EACH PREDICTOR HAS A SPECIFIED HARM

if (length(harm)>0 & pred.n!=length(harm)) {

stop("Number of harms specified must be the same as the number of predictors being checked.")

}

#INITIALIZING DEFAULT VALUES FOR PROBABILITES AND HARMS IF NOT SPECIFIED

if (length(harm)==0) {

harm=rep(0,pred.n)

}

if (length(probability)==0) {

probability=rep(TRUE,pred.n)

}

# THE PREDICTOR NAMES CANNOT BE EQUAL TO all OR none.

if (length(predictors[predictors=="all" | predictors=="none"])) {

stop("Prediction names cannot be equal to all or none.")

}

#CHECKING THAT EACH probability ELEMENT IS EQUAL TO T OR F,

#AND CHECKING THAT PROBABILITIES ARE BETWEEN 0 and 1

#IF NOT A PROB THEN CONVERTING WITH A COX REGRESSION

for(m in 1:pred.n) {

if (probability[m]!=TRUE & probability[m]!=FALSE) {

stop("Each element of probability vector must be TRUE or FALSE")

}

if (probability[m]==TRUE & (max(data[predictors[m]])>1 | min(data[predictors[m]])<0)) {

stop(paste(predictors[m],"must be between 0 and 1 OR sepcified as a non-probability in the probability option",sep=" "))

}

if(probability[m]==FALSE) {

model=NULL

pred=NULL

model=coxph(Surv(data.matrix(data[ttoutcome]),data.matrix(data[outcome])) ~ data.matrix(data[predictors[m]]))

surv.data=data.frame(0)

pred=data.frame(1-c(summary(survfit(model, newdata=surv.data), time=timepoint)$surv))

names(pred)=predictors[m]

data=cbind(data[names(data)!=predictors[m]],pred)

print(paste(predictors[m],"converted to a probability with Cox regression. Due to linearity and proportional hazards assumption, miscalibration may occur.",sep=" "))

}

}

######### CALCULATING NET BENEFIT #########

N=dim(data)[1]

# getting the probability of the event for all subjects

# this is used for the net benefit associated with treating all patients

if(cmprsk==FALSE) {

km.cuminc=survfit(Surv(data.matrix(data[ttoutcome]),data.matrix(data[outcome]))~1)

pd=1 - summary(km.cuminc, times=timepoint)$surv

} else {

require(cmprsk)

cr.cuminc=cuminc(data[[ttoutcome]],data[[outcome]])

pd=timepoints(cr.cuminc, times=timepoint)$est[1]

}

#creating dataset that is one line per threshold for the treat all and treat none strategies;

# CREATING DATAFRAME THAT IS ONE LINE PER THRESHOLD PER all AND none STRATEGY

nb=data.frame(seq(from=xstart, to=xstop, by=xby))

names(nb)="threshold"

interv=nb

error=NULL

nb["all"]=pd - (1-pd)*nb$threshold/(1-nb$threshold)

nb["none"]=0

# CYCLING THROUGH EACH PREDICTOR AND CALCULATING NET BENEFIT

for(m in 1:pred.n){

nb[predictors[m]]=NA

for(t in 1:length(nb$threshold)){

#calculating number of true and false postives;

px=sum(data[predictors[m]]>nb$threshold[t])/N

if (px==0){

error=rbind(error,paste(predictors[m],": No observations with risk greater than ",nb$threshold[t]*100,"%",sep=""))

break

} else {

#calculate risk using Kaplan Meier

if(cmprsk==FALSE) {

km.cuminc=survfit(Surv(data.matrix(data[data[predictors[m]]>nb$threshold[t],ttoutcome]),

data.matrix(data[data[predictors[m]]>nb$threshold[t],outcome]))~1)

pdgivenx=(1 - summary(km.cuminc, times=timepoint)$surv)

if(length(pdgivenx)==0){

error=rbind(error,paste(predictors[m],": No observations with risk greater than ",nb$threshold[t]*100,"% that have followup through the timepoint selected",sep=""))

break

}

#calculate risk using competing risk

} else {

cr.cuminc=cuminc(data[[ttoutcome]][data[[predictors[m]]]>nb$threshold[t]],data[[outcome]][data[[predictors[m]]]>nb$threshold[t]])

pdgivenx=timepoints(cr.cuminc, times=timepoint)$est[1]

if(is.na(pdgivenx)){

error=rbind(error,paste(predictors[m],": No observations with risk greater than ",nb$threshold[t]*100,"% that have followup through the timepoint selected",sep=""))

break

}

}

#calculating NB based on calculated risk

nb[t,predictors[m]]=pdgivenx*px - (1-pdgivenx)*px*nb$threshold[t]/(1-nb$threshold[t]) - harm[m]

}

}

interv[predictors[m]]=(nb[predictors[m]] - nb["all"])*interventionper/(interv$threshold/(1-interv$threshold))

}

if(length(error)>0){

print(paste(error,", and therefore net benefit not calculable in this range.",sep=""))

}

# CYCLING THROUGH EACH PREDICTOR AND SMOOTH NET BENEFIT AND INTERVENTIONS AVOIDED

for(m in 1:pred.n) {

if (smooth==TRUE){

lws=loess(data.matrix(nb[!is.na(nb[[predictors[m]]]),predictors[m]]) ~ data.matrix(nb[!is.na(nb[[predictors[m]]]),"threshold"]),span=loess.span)

nb[!is.na(nb[[predictors[m]]]),paste(predictors[m],"_sm",sep="")]=lws$fitted

lws=loess(data.matrix(interv[!is.na(nb[[predictors[m]]]),predictors[m]]) ~ data.matrix(interv[!is.na(nb[[predictors[m]]]),"threshold"]),span=loess.span)

interv[!is.na(nb[[predictors[m]]]),paste(predictors[m],"_sm",sep="")]=lws$fitted

}

}

# PLOTTING GRAPH IF REQUESTED

if (graph==TRUE) {

require(graphics)

# PLOTTING INTERVENTIONS AVOIDED IF REQUESTED

if(intervention==TRUE) {

# initialize the legend label, color, and width using the standard specs of the none and all lines

legendlabel <- NULL

legendcolor <- NULL

legendwidth <- NULL

legendpattern <- NULL

#getting maximum number of avoided interventions

ymax=max(interv[predictors],na.rm = TRUE)

#INITIALIZING EMPTY PLOT WITH LABELS

plot(x=nb$threshold, y=nb$all, type="n" ,xlim=c(xstart, xstop), ylim=c(ymin, ymax), xlab="Threshold probability", ylab=paste("Net reduction in interventions per",interventionper,"patients"))

#PLOTTING INTERVENTIONS AVOIDED FOR EACH PREDICTOR

for(m in 1:pred.n) {

if (smooth==TRUE){

lines(interv$threshold,data.matrix(interv[paste(predictors[m],"_sm",sep="")]),col=m,lty=2)

} else {

lines(interv$threshold,data.matrix(interv[predictors[m]]),col=m,lty=2)

}

# adding each model to the legend

legendlabel <- c(legendlabel, predictors[m])

legendcolor <- c(legendcolor, m)

legendwidth <- c(legendwidth, 1)

legendpattern <- c(legendpattern, 2)

}

} else {

# PLOTTING NET BENEFIT IF REQUESTED

# initialize the legend label, color, and width using the standard specs of the none and all lines

legendlabel <- c("None", "All")

legendcolor <- c(17, 8)

legendwidth <- c(2, 2)

legendpattern <- c(1, 1)

#getting maximum net benefit

ymax=max(nb[names(nb)!="threshold"],na.rm = TRUE)

# inializing new benfit plot with treat all option

plot(x=nb$threshold, y=nb$all, type="l", col=8, lwd=2 ,xlim=c(xstart, xstop), ylim=c(ymin, ymax), xlab="Threshold probability", ylab="Net benefit")

# adding treat none option

lines(x=nb$threshold, y=nb$none,lwd=2)

#PLOTTING net benefit FOR EACH PREDICTOR

for(m in 1:pred.n) {

if (smooth==TRUE){

lines(nb$threshold,data.matrix(nb[paste(predictors[m],"_sm",sep="")]),col=m,lty=2)

} else {

lines(nb$threshold,data.matrix(nb[predictors[m]]),col=m,lty=2)

}

# adding each model to the legend

legendlabel <- c(legendlabel, predictors[m])

legendcolor <- c(legendcolor, m)

legendwidth <- c(legendwidth, 1)

legendpattern <- c(legendpattern, 2)

}

}

# then add the legend

legend("topright", legendlabel, cex=0.8, col=legendcolor, lwd=legendwidth, lty=legendpattern)

}

#RETURNING RESULTS

results=list()

results$N=N

results$predictors=data.frame(cbind(predictors,harm,probability))

names(results$predictors)=c("predictor","harm.applied","probability")

results$interventions.avoided.per=interventionper

results$net.benefit=nb

results$interventions.avoided=interv

return(results)

}

data <- read.table("~/file.txt", header = T)

head(data)

# event time Age Weight loss Sex Grade Stage Score

# 2 1 455 82 15 Male 0 Stage1 90

# 3 0 1010 42 15 Male 2 Stage1 90

# 4 1 210 57 11 Male 0 Stage2 60

# 5 1 883 60 0 Male 2 Stage1 90

# 6 0 1022 74 0 Male 2 Stage2 80

# 7 1 310 68 10 Female 0 Stage3 60

nrow(data) ## 228

data <- na.omit(data)

nrow(data) ## 210

stdca(data = data, outcome = "event", ttoutcome = "time",

timepoint = 365, predictors = c("Age"), probability = F)
