# Supplementary material for: Classification of the mitochondrial ribosomal protein-associated molecular subtypes and identified a serological diagnostic biomarker in hepatocellular carcinoma
Source: Front Surg. 2023 Jan 6;9:1062659. doi: 10.3389/fsurg.2022.1062659 (PMC9853988; doi:10.3389/fsurg.2022.1062659)
Supplement: Supplementary file 1 [file Datasheet1.zip › GSEA.docx]

library(clusterProfiler)

library(org.Hs.eg.db) ## org.Mm.eg.db

library(msigdbr)

library(enrichplot)

data <- read.table("~/file.txt", header = T)

head(data)

# id logFC

# 1 NAT1 2.921874

# 2 ADH1B 2.104560

# 3 BIRC5 2.879421

# 4 AQP9 2.918583

# 5 BCL2A1 2.116960

# 6 BMP4 2.334144

gene_ids = bitr(geneID = data$id, fromType = "SYMBOL",

toType = "ENTREZID", OrgDb = "org.Hs.eg.db")

head(gene_ids)

# SYMBOL ENTREZID

# 1 NAT1 9

# 2 ADH1B 125

# 3 BIRC5 332

# 4 AQP9 366

# 5 BCL2A1 597

# 6 BMP4 652

gene_ids <- gene_ids[!duplicated(gene_ids$SYMBOL),]

tmp <- merge(gene_ids, data, by.x = "SYMBOL", by.y = "id")

geneList <- tmp$value

names(geneList) <- tmp$ENTREZID

geneList <- sort(geneList, decreasing = T)

## extract reference gene dataset

geneSet <- msigdbr(species = "Homo sapiens", category = "C2")

geneSet <- geneSet[, c("gs_name", "entrez_gene")]

set.seed(2021)

gsea <- GSEA(geneList = geneList,

TERM2GENE = geneSet,

pvalueCutoff = 0.3,

seed = T)

gsea

# #

# # Gene Set Enrichment Analysis

# #

# #...@organism UNKNOWN

# #...@setType UNKNOWN

# #...@geneList Named num [1:12444] 4.57 4.51 4.42 4.14 3.88 ...

# - attr(*, "names")= chr [1:12444] "4312" "8318" "10874" "55143" ...

# #...nPerm 1000

# #...pvalues adjusted by 'BH' with cutoff <0.3

# #...2251 enriched terms found

# 'data.frame': 2251 obs. of 11 variables:

# $ ID : chr "BONOME_OVARIAN_CANCER_SURVIVAL_SUBOPTIMAL_DEBULKING" "BROWNE_HCMV_INFECTION_48HR_DN" "REACTOME_SIGNALING_BY_RECEPTOR_TYROSINE_KINASES" "NAKAMURA_TUMOR_ZONE_PERIPHERAL_VS_CENTRAL_DN" ...

# $ Description : chr "BONOME_OVARIAN_CANCER_SURVIVAL_SUBOPTIMAL_DEBULKING" "BROWNE_HCMV_INFECTION_48HR_DN" "REACTOME_SIGNALING_BY_RECEPTOR_TYROSINE_KINASES" "NAKAMURA_TUMOR_ZONE_PERIPHERAL_VS_CENTRAL_DN" ...

# $ setSize : int 485 460 465 490 483 435 446 452 455 418 ...

# $ enrichmentScore: num -0.398 -0.388 -0.332 -0.351 -0.331 ...

# $ NES : num -1.78 -1.73 -1.48 -1.57 -1.48 ...

# $ pvalue : num 0.00127 0.00127 0.00127 0.00127 0.00128 ...

# $ p.adjust : num 0.0197 0.0197 0.0197 0.0197 0.0197 ...

# $ qvalues : num 0.0139 0.0139 0.0139 0.0139 0.0139 ...

# $ rank : num 2966 2921 2774 2566 2038 ...

# $ leading_edge : chr "tags=38%, list=24%, signal=30%" "tags=36%, list=23%, signal=29%" "tags=26%, list=22%, signal=21%" "tags=33%, list=21%, signal=27%" ...

# $ core_enrichment: chr "2909/2040/6383/6777/5168/55691/5789/49855/11080/23363/8613/554/5802/6939/8504/79058/7025/23515/9652/23411/6405/"| __truncated__ "5168/5789/8029/4008/7703/51230/64131/3425/4015/4548/158471/9180/9753/11099/5354/5273/5333/4026/4820/1639/7082/5"| __truncated__ "9101/26052/534/2065/2354/3709/7423/1215/2263/83464/26469/7057/4670/7072/1298/3915/5921/5441/200734/9846/9611/33"| __truncated__ "2114/120/9919/8714/7072/23499/10966/55667/51279/4601/57556/26959/55717/3321/7572/1992/5139/2581/26156/1806/2626"| __truncated__ ...

# #...Citation

# Guangchuang Yu, Li-Gen Wang, Yanyan Han and Qing-Yu He.

# clusterProfiler: an R package for comparing biological themes among

# gene clusters. OMICS: A Journal of Integrative Biology

# 2012, 16(5):284-287

gseaplot(gsea, geneSetID = 1)

gseaplot2(gsea, geneSetID = 1)

ridgeplot(gsea)
