# Supplementary material for: Classification of the mitochondrial ribosomal protein-associated molecular subtypes and identified a serological diagnostic biomarker in hepatocellular carcinoma
Source: Front Surg. 2023 Jan 6;9:1062659. doi: 10.3389/fsurg.2022.1062659 (PMC9853988; doi:10.3389/fsurg.2022.1062659)
Supplement: Supplementary file 1 [file Datasheet1.zip › heatmap.docx]

library(tidyverse)

library(ComplexHeatmap)

pheno <- read.table("~/pheno.txt", header = T)

head(pheno)

# sample group other

# 1 sample1 group2 other3

# 2 sample2 group2 other3

# 3 sample3 group2 other3

# 4 sample4 group2 other3

# 5 sample5 group2 other1

# 6 sample6 group2 other1

expr <- read.table("~/expr.txt", header = T, row.names = T)

head(expr[, 1:5])

# sample1 sample2 sample3 sample4 sample5

# Gene1 NA 2.2235 6.8363 3.0055 3.5615

# Gene2 -0.6216 1.0857 2.0330 1.1457 2.0632

# Gene3 -0.2429 5.5259 4.3451 5.0517 -4.9717

# Gene4 4.2098 0.6785 1.6226 0.1889 4.7902

# Gene5 -3.6314 1.6453 2.1495 1.3583 -0.0633

# Gene6 1.6555 -0.1864 -0.1317 -0.0404 2.2153

col = c("#4DBBD5", "#E64B35")

names(col) <- c("group1", "group2")

top <- HeatmapAnnotation(group = pheno$group, col = list(group = col))

Heatmap(as.matrix(t(scale(t(expr)))),

top_annotation = top, show_column_names = F)
