# Supplementary material for: Classification of the mitochondrial ribosomal protein-associated molecular subtypes and identified a serological diagnostic biomarker in hepatocellular carcinoma
Source: Front Surg. 2023 Jan 6;9:1062659. doi: 10.3389/fsurg.2022.1062659 (PMC9853988; doi:10.3389/fsurg.2022.1062659)
Supplement: Supplementary file 1 [file Datasheet1.zip › ic50.docx]

mypal = rep(pal_npg(alpha = 0.7)(7),2)

load('xxx.RData')

load('Clinical.RData')

comsamples=intersect(colnames(dt),c(paste0(rownames(clinical),'-01'),paste0(rownames(clinical),'-03'),paste0(rownames(clinical),'-06')))

data=log2(dt[,comsamples]+1)

set.seed(1234567)

#load('D:/ICGC/analysis/TCGA/TPM/TCGA_LUAD.RData')

result <- pRRopheticPredict(testMatrix = as.matrix(data.map),

drug = 'SB590885',tissueType = "all",

selection = 1)

cor_point=function(x,y,method='Pearson',top_col='#D55E00',right_col='#009E73'

,ylab='y expression',xlab='x expression',title=NULL

,marginal.type=c("histogram", "boxplot", "density", "violin", "densigram")[1]){

library(ggstatsplot)

dat=data.frame(X=x,Y=y)

tp='nonparametric'

if(method=='Pearson'|method=='pearson'){

tp='parametric'

}

g1=ggscatterstats(data = dat,

x = X,

y = Y

,type = tp

,xfill = top_col

,yfill = right_col

,xlab = xlab

,ylab=ylab

,marginal.type = marginal.type

,title = title)

return(g1)

}

dt.gene=as.numeric(data.map[dnaName,])

p.fin1=cor_point(x=dt.gene,y=as.numeric(result),top_col=mypal[1],right_col=mypal[2]

,ylab=paste0(paste0(drug.names,' IC50'))

,xlab=paste0('Log2 (',dnaName,' expression)')

,marginal.type='density',method = 'spearman')
