# Supplementary material for: Classification of the mitochondrial ribosomal protein-associated molecular subtypes and identified a serological diagnostic biomarker in hepatocellular carcinoma
Source: Front Surg. 2023 Jan 6;9:1062659. doi: 10.3389/fsurg.2022.1062659 (PMC9853988; doi:10.3389/fsurg.2022.1062659)
Supplement: Supplementary file 1 [file Datasheet1.zip › KEGG+GO.docx]

library(clusterProfiler)

library(org.Hs.eg.db) ## org.Mm.eg.db

gene_ids = bitr(geneID = gene_list, fromType = "SYMBOL",

toType = "ENTREZID", OrgDb = "org.Hs.eg.db")

head(gene_ids)

# SYMBOL ENTREZID

# 1 NAT1 9

# 2 ADH1B 125

# 3 BIRC5 332

# 4 AQP9 366

# 5 BCL2A1 597

# 6 BMP4 652

ego <- enrichGO(gene = gene_ids$ENTREZID, OrgDb = "org.Hs.eg.db",

keyType = "ENTREZID", ont = "BP")

# #

# # over-representation test

# #

# #...@organism Homo sapiens

# #...@ontology BP

# #...@keytype ENTREZID

# #...@gene chr [1:209] "9" "125" "332" "366" "597" "652" "730" "771" "776" "820" "890" "891" "983" "991" "1062" "1101" "1111" "1307" "1308" "1311" ...

# #...pvalues adjusted by 'BH' with cutoff <0.05

# #...187 enriched terms found

# 'data.frame': 187 obs. of 9 variables:

# $ ID : chr "GO:0140014" "GO:0000280" "GO:0048285" "GO:0000070" ...

# $ Description: chr "mitotic nuclear division" "nuclear division" "organelle fission" "mitotic sister chromatid segregation" ...

# $ GeneRatio : chr "32/196" "34/196" "35/196" "23/196" ...

# $ BgRatio : chr "264/18670" "407/18670" "449/18670" "151/18670" ...

# $ pvalue : num 7.53e-25 4.63e-21 1.10e-20 2.23e-20 2.61e-19 ...

# $ p.adjust : num 2.33e-21 7.16e-18 1.14e-17 1.72e-17 1.61e-16 ...

# $ qvalue : num 2.01e-21 6.16e-18 9.78e-18 1.48e-17 1.39e-16 ...

# $ geneID : chr "332/652/891/991/1062/1111/3832/3833/4085/4605/4751/6790/7272/9055/9212/9232/9319/9493/9787/10403/10460/11065/22"| __truncated__ "332/652/891/991/1062/1111/3832/3833/4085/4605/4751/6790/7153/7272/9055/9212/9232/9319/9493/9787/10403/10460/110"| __truncated__ "332/652/891/991/1062/1111/3832/3833/4085/4137/4605/4751/6790/7153/7272/9055/9212/9232/9319/9493/9787/10403/1046"| __truncated__ "891/991/1062/3833/4085/4751/7272/9055/9212/9232/9319/9493/9787/10403/10460/23397/24137/51203/55143/64151/81620/81930/146909" ...

# $ Count : int 32 34 35 23 24 28 20 24 17 19 ...

# #...Citation

# Guangchuang Yu, Li-Gen Wang, Yanyan Han and Qing-Yu He.

# clusterProfiler: an R package for comparing biological themes among

# gene clusters. OMICS: A Journal of Integrative Biology

# 2012, 16(5):284-287

#### enrichKEGG(gene = gene_ids$ENTREZID, organism = "hsa", keyType = "ENTREZID")

### dot plot

dotplot(ego)

### cnet plot

cnetplot(ego)
