# Supplementary material for: Classification of the mitochondrial ribosomal protein-associated molecular subtypes and identified a serological diagnostic biomarker in hepatocellular carcinoma
Source: Front Surg. 2023 Jan 6;9:1062659. doi: 10.3389/fsurg.2022.1062659 (PMC9853988; doi:10.3389/fsurg.2022.1062659)
Supplement: Supplementary file 1 [file Datasheet1.zip › LASSO-2.docx]

# library(tidyverse)

library(survival)

library(glmnet)

library(readxl)

## read data

data <- read_xlsx("~/file.xlsx")

head(data)

# event time Gene 1 Gene 2 Gene 3 Gene 4 Gene 5 Gene 6 Gene 7 Gene 8 Gene 9 Gene 10 Gene 11

# 1 1 306 -0.02228062 -2.7704356 -0.4670543 0.6597100 -0.5151171 0.02662303 0.9648682 -1.0649103 0.1261321 -1.2472454 0.2857966

# 2 1 455 -1.18321709 -0.3161180 0.6060377 -0.3035985 0.1487279 -1.21013808 1.2133766 -0.1951160 0.6075327 0.3840250 0.6994784

# 3 0 1010 -0.62297439 1.8458862 1.5176689 -0.8392173 -0.2736995 -1.91479449 0.9869319 -0.1859204 0.2624222 0.8615537 -0.5557269

# 4 1 210 -0.96143221 -0.1361290 0.7070270 -2.2407777 -0.1158965 -1.67839318 0.5813433 1.2488396 -0.4216805 -0.3247411 -0.1496818

# 5 1 883 -2.00905791 0.7544725 -1.3601112 0.7434566 1.2420130 0.37301567 0.6557969 -0.6545812 -0.7919030 -0.7085475 -2.8072731

# 6 0 1022 0.79356585 -0.2366209 -0.5012338 0.9380560 -1.2196590 -1.62508198 0.3280815 1.0461292 -0.6751357 1.4510194 -0.7491115

set.seed(2021)

cvfit = cv.glmnet(x = as.matrix(data[,c(-1, -2)]),

y = Surv(time = data$time, event = data$event), family = "cox",

alpha = 1)

# cvfit

# Call: cv.glmnet(x = as.matrix(data[, c(-1, -2)]), y = Surv(time = data$time, event = data$event), family = "cox", alpha = 1)

#

# Measure: Partial Likelihood Deviance

#

# Lambda Index Measure SE Nonzero

# min 0.09095 7 10.16 0.1983 4

# 1se 0.15894 1 10.17 0.1893 0

## Check Coefficients

# coef(cvfit, s = "lambda.1se")

coef.min = coef(cvfit, s = "lambda.min")

## riskscore

as.matrix(dat1[,c(-1,-2)]) %*% as.matrix(coef.min)

## plot

plot(cvfit)

plot(cvfit$glmnet.fit, xvar = "norm")

plot(cvfit$glmnet.fit, xvar = "lambda")
