# Supplementary material for: Classification of the mitochondrial ribosomal protein-associated molecular subtypes and identified a serological diagnostic biomarker in hepatocellular carcinoma
Source: Front Surg. 2023 Jan 6;9:1062659. doi: 10.3389/fsurg.2022.1062659 (PMC9853988; doi:10.3389/fsurg.2022.1062659)
Supplement: Supplementary file 1 [file Datasheet1.zip › nomogram.docx]

library(tidyverse)

library(survival)

library(readxl)

library(rms)

## read data

data <- read_xlsx("~/file.xlsx")

## tidy data

# data$event <- as.numeric(data$event)

# data$time <- as.numeric(data$time)

### numeric

data$Age <- as.numeric(data$Age)

data$Score <- as.numeric(data$Score)

### factor

data$Sex <- factor(data$Sex, levels = c("Male", "Female"))

data$Grade <- factor(data$Grade, levels = c("0", "1", "2"))

data$Stage <- factor(data$Stage, levels = c("Stage1", "Stage2", "Stage3", "Stage4"))

colnames(data)[4] <- "Weight_loss"

## summary

fit <- survfit(Surv(time, event) ~ Sex, data = data)

fit

# Call: survfit(formula = Surv(time, event) ~ Sex, data = data)

#

# n events median 0.95LCL 0.95UCL

# Sex=Male 138 112 270 212 310

# Sex=Female 90 53 426 348 550

fit <- coxph(Surv(time = time, event = event) ~ Age + Weight_loss + Sex + Grade + Stage + Score, data = data)

summary(fit)

# Call:

# coxph(formula = Surv(time = time, event = event) ~ Age + Weight_loss +

# Sex + Grade + Stage + Score, data = data)

#

# n= 210, number of events= 148

# (18 observations deleted due to missingness)

#

# coef exp(coef) se(coef) z Pr(>|z|)

# Age 0.012963 1.013047 0.009363 1.384 0.16622

# Weight_loss -0.013179 0.986908 0.007049 -1.870 0.06153 .

# SexFemale -0.648482 0.522839 0.181763 -3.568 0.00036 ***

# Grade1 0.247066 1.280264 0.215206 1.148 0.25095

# Grade2 -0.167493 0.845782 0.243006 -0.689 0.49066

# StageStage2 0.447155 1.563856 0.211797 2.111 0.03475 *

# StageStage3 0.791586 2.206894 0.299925 2.639 0.00831 **

# StageStage4 2.083022 8.028691 1.056227 1.972 0.04859 *

# Score -0.013471 0.986619 0.007642 -1.763 0.07795 .

# ---

# Signif. codes: 0 鈥�***鈥� 0.001 鈥�**鈥� 0.01 鈥�*鈥� 0.05 鈥�.鈥� 0.1 鈥� 鈥� 1

#

# exp(coef) exp(-coef) lower .95 upper .95

# Age 1.0130 0.9871 0.9946 1.0318

# Weight_loss 0.9869 1.0133 0.9734 1.0006

# SexFemale 0.5228 1.9126 0.3661 0.7466

# Grade1 1.2803 0.7811 0.8397 1.9520

# Grade2 0.8458 1.1823 0.5253 1.3618

# StageStage2 1.5639 0.6394 1.0326 2.3685

# StageStage3 2.2069 0.4531 1.2260 3.9726

# StageStage4 8.0287 0.1246 1.0129 63.6366

# Score 0.9866 1.0136 0.9720 1.0015

#

# Concordance= 0.665 (se = 0.026 )

# Likelihood ratio test= 38.77 on 9 df, p=1e-05

# Wald test = 36.84 on 9 df, p=3e-05

# Score (logrank) test = 39.94 on 9 df, p=8e-06

dat1 = datadist(data)

options(datadist = "dat1")

cph1 <- cph(formula = Surv(time = time, event = event) ~ Age + Weight_loss + Sex + Grade + Stage + Score,

data=data, x=T, y=T, surv = T)

surv <- Survival(cph1)

surv1 <- function(x) surv(365*1,x)

surv2 <- function(x) surv(365*2,x)

fit <- nomogram(cph1, fun = list(surv1, surv2), lp=T,

funlabel = c("1-Year", "2-Year"))

plot(fit)
