# Supplementary material for: Classification of the mitochondrial ribosomal protein-associated molecular subtypes and identified a serological diagnostic biomarker in hepatocellular carcinoma
Source: Front Surg. 2023 Jan 6;9:1062659. doi: 10.3389/fsurg.2022.1062659 (PMC9853988; doi:10.3389/fsurg.2022.1062659)
Supplement: Supplementary file 1 [file Datasheet1.zip › PCA.docx]

library(tidyverse)

library(ggplot2)

data1 <- read.table("~/group.txt", header = T)

data2 <- read.table("~/expr.txt", header = T)

head(data1)

# sample_id group condition cluster

# 1 GSM831759 group1 control A

# 2 GSM831760 group1 control A

# 3 GSM831761 group1 control B

# 4 GSM831762 group1 control B

# 5 GSM831763 group1 control C

# 6 GSM831846 group2 treatmentA C

head(data2)

# GSM831759 GSM831760 GSM831761 GSM831762 GSM831763 GSM831846 GSM831847 GSM831848 GSM831849 GSM831850

# 1 14.62941 14.61866 14.68541 14.50261 14.56889 14.62878 14.65677 14.42425 14.63458 14.56247

# 2 14.43636 14.43300 14.44328 14.43701 14.41977 14.46710 14.48100 14.32143 14.43067 14.45823

# 3 14.29299 14.25202 14.48144 13.59690 14.55496 14.56642 14.58414 14.30457 14.62427 14.67283

# 4 14.27772 14.22394 14.23565 14.20099 14.21491 14.22601 14.24041 14.10793 14.27520 14.25602

# 5 14.14295 14.30087 14.30292 14.03515 14.09056 14.19274 14.17352 14.06926 14.15766 14.17658

# 6 14.22529 14.05115 14.18939 14.20989 14.15507 14.11961 13.90419 13.84729 14.22100 14.19716

pca = prcomp(t(data2))

data1 <- data.frame("PC1" = pca$x[,1],

"PC2" = pca$x[,2]) %>%

rownames_to_column("sample_id") %>%

left_join(., dat_attrs, by = "sample_id")

ggplot() +

geom_hline(yintercept = 0, linetype = 2, size = 0.75*0.47) +

geom_vline(xintercept = 0, linetype = 2, size = 0.75*0.47) +

geom_point(data = data1, aes(x = PC1, y = PC2, colour = group, fill = group))
