# Supplementary material for: Classification of the mitochondrial ribosomal protein-associated molecular subtypes and identified a serological diagnostic biomarker in hepatocellular carcinoma
Source: Front Surg. 2023 Jan 6;9:1062659. doi: 10.3389/fsurg.2022.1062659 (PMC9853988; doi:10.3389/fsurg.2022.1062659)
Supplement: Supplementary file 2 [file Datasheet2.zip › FigureS1.docx]

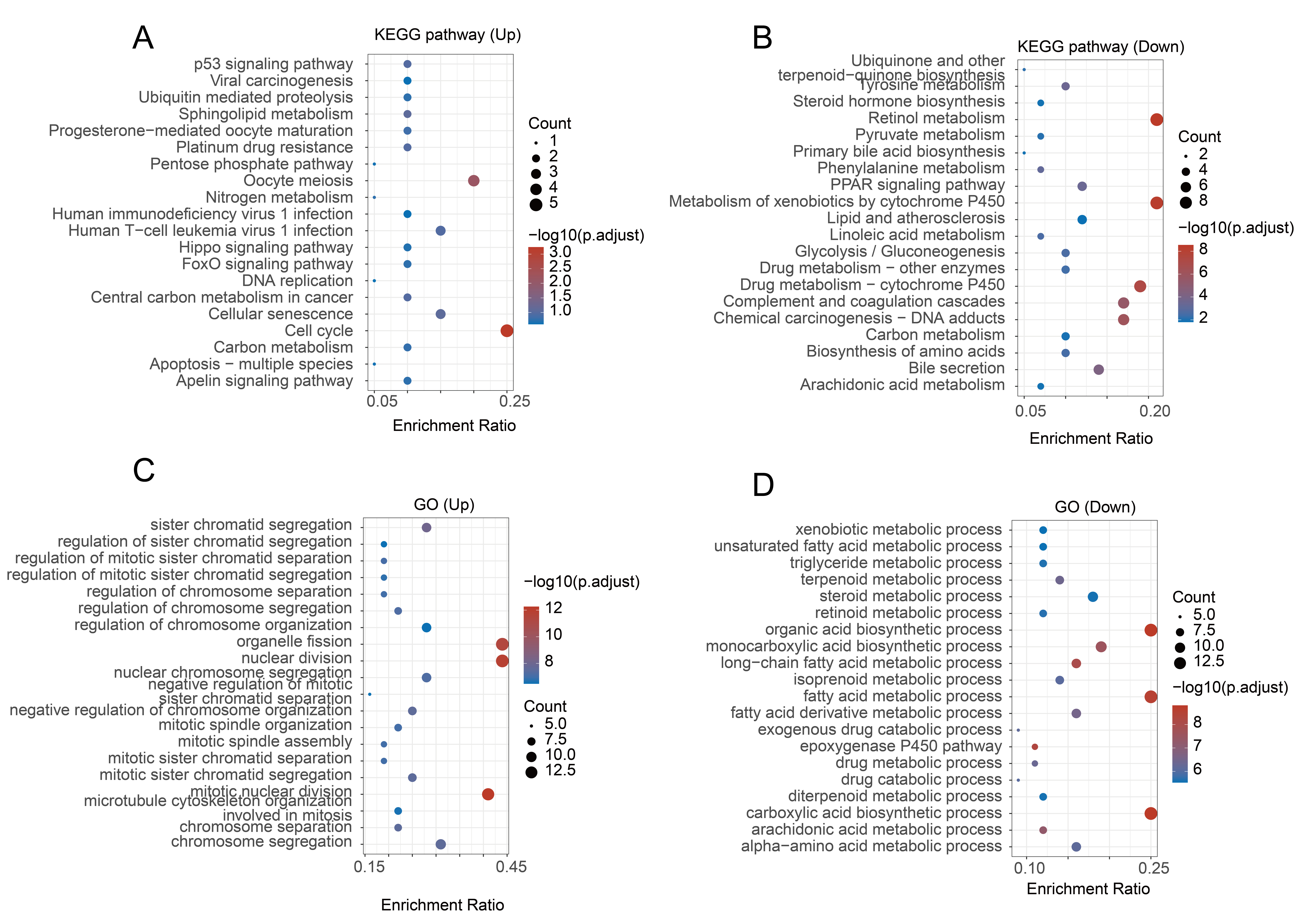


**Figure S1**

GO and KEGG analysis of DEGs in two clusters. (A-B) KEGG analysis of DEGs. (C-D) GO analysis of DEGs.
