# Supplementary material for: Classification of the mitochondrial ribosomal protein-associated molecular subtypes and identified a serological diagnostic biomarker in hepatocellular carcinoma
Source: Front Surg. 2023 Jan 6;9:1062659. doi: 10.3389/fsurg.2022.1062659 (PMC9853988; doi:10.3389/fsurg.2022.1062659)
Supplement: Supplementary file 2 [file Datasheet2.zip › TableS1.docx]

**Table S1** OS prognosis of MRPs in HCC patients

| Genes | p.value | HR | Low 95%CI | High 95%CI |
| --- | --- | --- | --- | --- |
| MRPL37 | 0.000026552 | 2.151996814 | 1.505089366 | 3.076953696 |
| MRPL36 | 0.000081372 | 2.046918219 | 1.433360253 | 2.923113145 |
| MRPL11 | 0.000122662 | 2.006732105 | 1.406457150 | 2.863204002 |
| MRPL53 | 0.000180000 | 1.910000000 | 1.350000000 | 2.690000000 |
| MRPL48 | 0.000189808 | 1.961352972 | 1.376957542 | 2.793772041 |
| MRPS7 | 0.000510742 | 1.877638445 | 1.316110543 | 2.678746210 |
| MRPL1 | 0.000564151 | 1.857391471 | 1.306357646 | 2.640856497 |
| MRPL3 | 0.000692610 | 1.843062817 | 1.294577065 | 2.623930733 |
| MRPL18 | 0.000776014 | 1.821312768 | 1.283958778 | 2.583556619 |
| MRPS12 | 0.000899079 | 1.810286094 | 1.275268304 | 2.569761775 |
| MRPS15 | 0.001186690 | 1.792474812 | 1.259591872 | 2.550799211 |
| MRPL58 | 0.001319139 | 1.782661217 | 1.252726203 | 2.536772208 |
| MRPL9 | 0.002231353 | 1.730564807 | 1.217600817 | 2.459635794 |
| MRPL17 | 0.003279928 | 1.689042474 | 1.190950400 | 2.395451968 |
| MRPL10 | 0.003309842 | 1.682778823 | 1.189080869 | 2.381456671 |
| MRPS5 | 0.003371952 | 1.692098914 | 1.190445742 | 2.405148452 |
| MRPL52 | 0.004749754 | 1.654149420 | 1.166407808 | 2.345843612 |
| MRPS16 | 0.005368372 | 1.648035666 | 1.159372570 | 2.342665012 |
| MRPL47 | 0.006750573 | 1.617950619 | 1.142282136 | 2.291696703 |
| MRPS23 | 0.008911756 | 1.588955479 | 1.123055177 | 2.248134878 |
| MRPL50 | 0.011921269 | 1.560757727 | 1.103151811 | 2.208186270 |
| MRPL42 | 0.011968495 | 1.559905753 | 1.102806893 | 2.206466040 |
| MRPL33 | 0.012739865 | 1.556672011 | 1.098931138 | 2.205076976 |
| MRPL45 | 0.014199772 | 1.547100277 | 1.091542422 | 2.192786298 |
| MRPS6 | 0.017043496 | 1.535866452 | 1.079592242 | 2.184978428 |
| MRPL35 | 0.017489628 | 1.528911465 | 1.077212041 | 2.170018695 |
| MRPL30 | 0.017867247 | 1.524056575 | 1.075365898 | 2.159961041 |
| MRPS26 | 0.019936126 | 1.510744366 | 1.067331158 | 2.138369634 |
| MRPS14 | 0.022906455 | 1.496179494 | 1.057379052 | 2.117077197 |
| MRPL38 | 0.023656827 | 1.498266156 | 1.055576959 | 2.126610908 |
| MRPS30 | 0.024252729 | 1.493944528 | 1.053614246 | 2.118299238 |
| MRPL51 | 0.024342811 | 1.489705006 | 1.052993400 | 2.107535531 |
| MRPL22 | 0.031111117 | 1.465352724 | 1.035294318 | 2.074056207 |
| MRPL27 | 0.034859911 | 1.457995946 | 1.027171446 | 2.069520319 |
| MRPL14 | 0.035780625 | 1.448920066 | 1.024936448 | 2.048292224 |
| MRPL21 | 0.045794349 | 1.431864003 | 1.006730157 | 2.036528368 |
| MRPS9 | 0.045920804 | 1.429064374 | 1.006487804 | 2.029060836 |
| MRPL54 | 0.046603506 | 0.701473209 | 0.494695214 | 0.994682480 |
| MRPS25 | 0.050740790 | 1.411310344 | 0.998889911 | 1.994010417 |
| MRPS27 | 0.056914133 | 1.399091388 | 0.990172310 | 1.976884925 |
| MRPS34 | 0.058389541 | 1.401716244 | 0.988081722 | 1.988508019 |
| MRPL12 | 0.061156012 | 1.397577481 | 0.984461246 | 1.984052521 |
| MRPS35 | 0.063590626 | 1.390270837 | 0.981535492 | 1.969213559 |
| MRPL13 | 0.064306493 | 1.386307544 | 0.980780550 | 1.959509297 |
| MRPS21 | 0.070536681 | 1.376500018 | 0.973583198 | 1.946163723 |
| MRPL44 | 0.088457016 | 1.349912593 | 0.955851827 | 1.906429382 |
| MRPS22 | 0.088815733 | 1.350031148 | 0.955467723 | 1.907530792 |
| MRPS10 | 0.093542747 | 1.347046372 | 0.950974495 | 1.908078436 |
| MRPL28 | 0.098079546 | 1.339479912 | 0.947415027 | 1.893791405 |
| MRPL15 | 0.104418429 | 1.331521356 | 0.942447363 | 1.881218187 |
| MRPS31 | 0.113958098 | 0.754020155 | 0.531301538 | 1.070101164 |
| MRPS18B | 0.175343546 | 1.271450887 | 0.898378700 | 1.799449784 |
| MRPS28 | 0.189113454 | 0.789757237 | 0.555267877 | 1.123271342 |
| MRPS33 | 0.200748069 | 1.252528433 | 0.887126572 | 1.768437025 |
| MRPL24 | 0.237174143 | 1.232019408 | 0.871701549 | 1.741274665 |
| MRPL4 | 0.241627073 | 1.229448287 | 0.870058536 | 1.737288962 |
| MRPL49 | 0.280691102 | 1.209121570 | 0.856316068 | 1.707284293 |
| MRPL55 | 0.282742798 | 1.208296172 | 0.855536241 | 1.706508234 |
| MRPL34 | 0.298639872 | 1.200892849 | 0.850313386 | 1.696014266 |
| MRPS18A | 0.305547197 | 1.197642600 | 0.848195640 | 1.691057736 |
| MRPS18C | 0.307870233 | 0.833344427 | 0.587004795 | 1.183061774 |
| MRPS2 | 0.358455487 | 1.175532865 | 0.832399663 | 1.660113019 |
| MRPS17 | 0.434062850 | 1.147345113 | 0.813072646 | 1.619044517 |
| MRPL32 | 0.436481073 | 1.146963977 | 0.812005027 | 1.620096332 |
| MRPL43 | 0.436650235 | 1.146529334 | 0.812369366 | 1.618142644 |
| MRPL2 | 0.445637203 | 1.143406937 | 0.810288638 | 1.613473721 |
| MRPL20 | 0.468711947 | 1.135832240 | 0.804804618 | 1.603016247 |
| MRPL46 | 0.489245318 | 0.884446985 | 0.624485419 | 1.252625675 |
| MRPL41 | 0.522952426 | 1.120347691 | 0.790549167 | 1.587730406 |
| MRPL23 | 0.593902066 | 1.098364001 | 0.777975947 | 1.550695089 |
| MRPL19 | 0.605320838 | 1.094982568 | 0.776148817 | 1.544789862 |
| MRPL39 | 0.645604819 | 1.084380147 | 0.767785754 | 1.531521387 |
| MRPS11 | 0.646024761 | 0.921841343 | 0.651375299 | 1.304611123 |
| MRPL16 | 0.678314701 | 1.075642907 | 0.762115275 | 1.518153094 |
| MRPL57 | 0.822695088 | 1.040188399 | 0.736950184 | 1.468202233 |
| MRPL40 | 0.865631187 | 0.970684956 | 0.687711358 | 1.370094113 |
| MRPS24 | 0.872084169 | 0.971960839 | 0.687542647 | 1.374035308 |
| MRPS36 | 0.969525143 | 1.006743965 | 0.713123023 | 1.421260257 |
| MRPL56 | 0.992688219 | 1.001612364 | 0.709670406 | 1.413652476 |
