# Supplementary material for: Classification of the mitochondrial ribosomal protein-associated molecular subtypes and identified a serological diagnostic biomarker in hepatocellular carcinoma
Source: Front Surg. 2023 Jan 6;9:1062659. doi: 10.3389/fsurg.2022.1062659 (PMC9853988; doi:10.3389/fsurg.2022.1062659)
Supplement: Supplementary file 2 [file Datasheet2.zip › TableS2.docx]

**Table S2** Clusters of HCC patients based on MRPs levels

|  | Cluster |
| --- | --- |
| TCGA-G3-A25X-01 | C1 |
| TCGA-DD-AADS-01 | C2 |
| TCGA-G3-AAV3-01 | C2 |
| TCGA-T1-A6J8-01 | C2 |
| TCGA-2Y-A9GU-01 | C2 |
| TCGA-2Y-A9HB-01 | C2 |
| TCGA-DD-A73E-01 | C1 |
| TCGA-UB-A7MB-01 | C1 |
| TCGA-DD-A73A-01 | C2 |
| TCGA-DD-AAEG-01 | C1 |
| TCGA-ED-A8O6-01 | C2 |
| TCGA-G3-AAV7-01 | C1 |
| TCGA-CC-A7IJ-01 | C1 |
| TCGA-2Y-A9H9-01 | C2 |
| TCGA-CC-5261-01 | C1 |
| TCGA-RC-A7SH-01 | C2 |
| TCGA-DD-A1EI-01 | C1 |
| TCGA-G3-A3CJ-01 | C2 |
| TCGA-GJ-A9DB-01 | C1 |
| TCGA-ED-A7PY-01 | C2 |
| TCGA-K7-A5RG-01 | C2 |
| TCGA-G3-A7M5-01 | C2 |
| TCGA-DD-AADN-01 | C2 |
| TCGA-CC-A8HS-01 | C1 |
| TCGA-WQ-AB4B-01 | C2 |
| TCGA-FV-A23B-01 | C2 |
| TCGA-FV-A3R3-01 | C2 |
| TCGA-G3-AAV0-01 | C2 |
| TCGA-5C-A9VG-01 | C1 |
| TCGA-BC-4072-01 | C2 |
| TCGA-DD-AAVV-01 | C1 |
| TCGA-DD-AADB-01 | C1 |
| TCGA-DD-AAVQ-01 | C2 |
| TCGA-CC-5258-01 | C1 |
| TCGA-EP-A3JL-01 | C2 |
| TCGA-DD-A73C-01 | C2 |
| TCGA-G3-AAV1-01 | C2 |
| TCGA-DD-AAEE-01 | C1 |
| TCGA-G3-A25T-01 | C1 |
| TCGA-LG-A9QC-01 | C2 |
| TCGA-ED-A82E-01 | C1 |
| TCGA-DD-AACF-01 | C2 |
| TCGA-QA-A7B7-01 | C1 |
| TCGA-DD-AAC9-01 | C2 |
| TCGA-DD-AAE6-01 | C2 |
| TCGA-DD-A3A2-01 | C2 |
| TCGA-DD-A39Y-01 | C1 |
| TCGA-BC-A112-01 | C1 |
| TCGA-ZP-A9CZ-01 | C1 |
| TCGA-CC-A3MB-01 | C1 |
| TCGA-DD-AAE1-01 | C2 |
| TCGA-EP-A3RK-01 | C1 |
| TCGA-G3-A3CG-01 | C2 |
| TCGA-DD-AA3A-01 | C1 |
| TCGA-BC-4073-01 | C2 |
| TCGA-DD-AACK-01 | C2 |
| TCGA-XR-A8TE-01 | C2 |
| TCGA-G3-A25V-01 | C2 |
| TCGA-DD-A4NA-01 | C2 |
| TCGA-DD-AAD3-01 | C2 |
| TCGA-DD-A118-01 | C2 |
| TCGA-CC-A9FV-01 | C2 |
| TCGA-ED-A459-01 | C2 |
| TCGA-RC-A7SB-01 | C2 |
| TCGA-WQ-A9G7-01 | C2 |
| TCGA-DD-AAEA-01 | C2 |
| TCGA-G3-A7M7-01 | C2 |
| TCGA-BC-A10T-01 | C2 |
| TCGA-DD-AADP-01 | C2 |
| TCGA-DD-AACT-01 | C2 |
| TCGA-UB-A7MA-01 | C1 |
| TCGA-DD-A73D-01 | C2 |
| TCGA-DD-A4NB-01 | C2 |
| TCGA-2Y-A9GT-01 | C2 |
| TCGA-DD-AADL-01 | C2 |
| TCGA-DD-A1EG-01 | C2 |
| TCGA-DD-A4ND-01 | C2 |
| TCGA-2Y-A9H6-01 | C2 |
| TCGA-DD-AAW1-01 | C2 |
| TCGA-DD-A1EA-01 | C2 |
| TCGA-ED-A66Y-01 | C2 |
| TCGA-DD-AACH-01 | C1 |
| TCGA-XR-A8TC-01 | C2 |
| TCGA-DD-A3A4-01 | C2 |
| TCGA-DD-A11A-01 | C2 |
| TCGA-DD-AADF-01 | C2 |
| TCGA-ZP-A9D0-01 | C2 |
| TCGA-UB-AA0V-01 | C2 |
| TCGA-MR-A520-01 | C2 |
| TCGA-RC-A6M5-01 | C2 |
| TCGA-EP-A2KA-01 | C2 |
| TCGA-DD-AAE7-01 | C2 |
| TCGA-DD-AAE4-01 | C2 |
| TCGA-DD-A4NF-01 | C2 |
| TCGA-BW-A5NP-01 | C1 |
| TCGA-RC-A6M3-01 | C2 |
| TCGA-DD-AACP-01 | C1 |
| TCGA-DD-AAE0-01 | C1 |
| TCGA-BC-A8YO-01 | C1 |
| TCGA-DD-A3A9-01 | C1 |
| TCGA-CC-A7II-01 | C1 |
| TCGA-DD-AAD1-01 | C2 |
| TCGA-XR-A8TF-01 | C2 |
| TCGA-G3-A6UC-01 | C2 |
| TCGA-DD-A1ED-01 | C2 |
| TCGA-CC-5262-01 | C1 |
| TCGA-ZS-A9CF-01 | C2 |
| TCGA-FV-A3I1-01 | C2 |
| TCGA-DD-AAW0-01 | C2 |
| TCGA-DD-AAD8-01 | C2 |
| TCGA-ZP-A9D4-01 | C2 |
| TCGA-RC-A7SK-01 | C1 |
| TCGA-DD-AAC8-01 | C1 |
| TCGA-GJ-A3OU-01 | C2 |
| TCGA-KR-A7K8-01 | C2 |
| TCGA-CC-A1HT-01 | C1 |
| TCGA-BC-A69H-01 | C1 |
| TCGA-DD-AAVR-01 | C2 |
| TCGA-DD-AAE3-01 | C2 |
| TCGA-DD-A4NN-01 | C1 |
| TCGA-DD-AACC-01 | C2 |
| TCGA-DD-AADI-01 | C1 |
| TCGA-DD-AAEB-01 | C2 |
| TCGA-2Y-A9H1-01 | C2 |
| TCGA-DD-AACB-01 | C1 |
| TCGA-WX-AA46-01 | C2 |
| TCGA-DD-A1EJ-01 | C1 |
| TCGA-EP-A2KB-01 | C2 |
| TCGA-ED-A66X-01 | C2 |
| TCGA-DD-AACW-01 | C1 |
| TCGA-NI-A4U2-01 | C2 |
| TCGA-ED-A97K-01 | C2 |
| TCGA-G3-A3CK-01 | C2 |
| TCGA-DD-AADW-01 | C1 |
| TCGA-DD-AAVP-01 | C1 |
| TCGA-DD-A39W-01 | C2 |
| TCGA-DD-A4NR-01 | C1 |
| TCGA-DD-AACU-01 | C1 |
| TCGA-KR-A7K7-01 | C1 |
| TCGA-DD-AACQ-01 | C2 |
| TCGA-DD-A11B-01 | C2 |
| TCGA-DD-A4NS-01 | C2 |
| TCGA-DD-A1EH-01 | C2 |
| TCGA-DD-AAW3-01 | C2 |
| TCGA-DD-AADA-01 | C2 |
| TCGA-UB-A7MF-01 | C1 |
| TCGA-DD-A119-01 | C2 |
| TCGA-2Y-A9H8-01 | C2 |
| TCGA-K7-A6G5-01 | C2 |
| TCGA-WJ-A86L-01 | C2 |
| TCGA-ZS-A9CG-01 | C2 |
| TCGA-O8-A75V-01 | C2 |
| TCGA-ED-A7PZ-01 | C1 |
| TCGA-DD-A1EL-01 | C1 |
| TCGA-G3-A5SL-01 | C2 |
| TCGA-DD-A1EE-01 | C2 |
| TCGA-CC-5263-01 | C1 |
| TCGA-G3-A3CI-01 | C2 |
| TCGA-ED-A5KG-01 | C1 |
| TCGA-DD-AAVS-01 | C1 |
| TCGA-DD-A4NO-01 | C2 |
| TCGA-CC-A3MA-01 | C1 |
| TCGA-NI-A8LF-01 | C2 |
| TCGA-5C-A9VH-01 | C2 |
| TCGA-DD-A4NE-01 | C2 |
| TCGA-FV-A4ZQ-01 | C1 |
| TCGA-K7-A5RF-01 | C2 |
| TCGA-DD-A1EB-01 | C2 |
| TCGA-DD-AAVU-01 | C1 |
| TCGA-DD-AADU-01 | C2 |
| TCGA-2Y-A9H0-01 | C2 |
| TCGA-GJ-A6C0-01 | C2 |
| TCGA-ED-A4XI-01 | C2 |
| TCGA-G3-A7M8-01 | C2 |
| TCGA-CC-A3M9-01 | C1 |
| TCGA-FV-A2QR-01 | C2 |
| TCGA-MR-A8JO-01 | C2 |
| TCGA-DD-A3A6-01 | C2 |
| TCGA-G3-AAV4-01 | C1 |
| TCGA-DD-A11D-01 | C2 |
| TCGA-BD-A3EP-01 | C2 |
| TCGA-EP-A12J-01 | C2 |
| TCGA-4R-AA8I-01 | C1 |
| TCGA-CC-A7IF-01 | C2 |
| TCGA-KR-A7K0-01 | C2 |
| TCGA-CC-A5UE-01 | C1 |
| TCGA-HP-A5MZ-01 | C2 |
| TCGA-CC-A8HU-01 | C1 |
| TCGA-DD-AADO-01 | C2 |
| TCGA-DD-AAD5-01 | C1 |
| TCGA-LG-A6GG-01 | C2 |
| TCGA-G3-A5SI-01 | C2 |
| TCGA-RG-A7D4-01 | C1 |
| TCGA-BW-A5NQ-01 | C1 |
| TCGA-KR-A7K2-01 | C2 |
| TCGA-DD-AADM-01 | C2 |
| TCGA-XR-A8TG-01 | C2 |
| TCGA-DD-AACD-01 | C2 |
| TCGA-3K-AAZ8-01 | C1 |
| TCGA-DD-AAD0-01 | C2 |
| TCGA-DD-AADJ-01 | C2 |
| TCGA-ES-A2HS-01 | C2 |
| TCGA-HP-A5N0-01 | C2 |
| TCGA-2V-A95S-01 | C2 |
| TCGA-ED-A7XP-01 | C2 |
| TCGA-G3-A7M9-01 | C1 |
| TCGA-G3-A3CH-01 | C2 |
| TCGA-G3-A25S-01 | C1 |
| TCGA-2Y-A9GW-01 | C2 |
| TCGA-BC-A217-01 | C1 |
| TCGA-DD-AACX-01 | C2 |
| TCGA-CC-A5UD-01 | C1 |
| TCGA-EP-A26S-01 | C2 |
| TCGA-MI-A75E-01 | C2 |
| TCGA-DD-AACO-01 | C2 |
| TCGA-G3-A5SK-01 | C2 |
| TCGA-BC-A110-01 | C2 |
| TCGA-G3-A5SM-01 | C2 |
| TCGA-ED-A8O5-01 | C2 |
| TCGA-FV-A3R2-01 | C1 |
| TCGA-DD-A116-01 | C2 |
| TCGA-DD-A39X-01 | C1 |
| TCGA-2Y-A9H2-01 | C1 |
| TCGA-CC-A9FS-01 | C2 |
| TCGA-XR-A8TD-01 | C2 |
| TCGA-ZP-A9D2-01 | C1 |
| TCGA-DD-A4NK-01 | C2 |
| TCGA-FV-A4ZP-01 | C2 |
| TCGA-DD-AAE2-01 | C2 |
| TCGA-DD-A39Z-01 | C2 |
| TCGA-5R-AA1D-01 | C2 |
| TCGA-2Y-A9H7-01 | C2 |
| TCGA-CC-5264-01 | C1 |
| TCGA-UB-A7ME-01 | C2 |
| TCGA-UB-A7MD-01 | C2 |
| TCGA-RC-A6M6-01 | C1 |
| TCGA-DD-A3A8-01 | C2 |
| TCGA-ED-A7PX-01 | C1 |
| TCGA-DD-AACZ-01 | C1 |
| TCGA-DD-A39V-01 | C2 |
| TCGA-ZS-A9CE-01 | C2 |
| TCGA-5R-AA1C-01 | C1 |
| TCGA-G3-A25Z-01 | C2 |
| TCGA-5R-AAAM-01 | C2 |
| TCGA-ES-A2HT-01 | C2 |
| TCGA-DD-A3A7-01 | C2 |
| TCGA-BD-A2L6-01 | C2 |
| TCGA-DD-AAE9-01 | C2 |
| TCGA-CC-A7IH-01 | C2 |
| TCGA-DD-AACL-01 | C1 |
| TCGA-CC-5259-01 | C2 |
| TCGA-G3-A7M6-01 | C1 |
| TCGA-DD-AAD6-01 | C2 |
| TCGA-FV-A2QQ-01 | C1 |
| TCGA-2Y-A9GX-01 | C2 |
| TCGA-BD-A3ER-01 | C2 |
| TCGA-DD-AADC-01 | C1 |
| TCGA-BC-A10Z-01 | C2 |
| TCGA-5C-AAPD-01 | C2 |
| TCGA-DD-AAED-01 | C2 |
| TCGA-DD-AACI-01 | C1 |
| TCGA-G3-A25Y-01 | C1 |
| TCGA-ZP-A9CY-01 | C2 |
| TCGA-PD-A5DF-01 | C1 |
| TCGA-DD-A114-01 | C2 |
| TCGA-DD-AAVY-01 | C2 |
| TCGA-BC-A10S-01 | C2 |
| TCGA-CC-A123-01 | C2 |
| TCGA-MI-A75H-01 | C2 |
| TCGA-DD-AADG-01 | C2 |
| TCGA-BC-A10U-01 | C2 |
| TCGA-CC-A7IG-01 | C1 |
| TCGA-DD-A3A3-01 | C2 |
| TCGA-2Y-A9GZ-01 | C2 |
| TCGA-K7-AAU7-01 | C2 |
| TCGA-BW-A5NO-01 | C2 |
| TCGA-DD-AAVX-01 | C2 |
| TCGA-DD-A4NJ-01 | C2 |
| TCGA-DD-AACS-01 | C2 |
| TCGA-UB-AA0U-01 | C2 |
| TCGA-CC-A9FW-01 | C1 |
| TCGA-CC-A8HT-01 | C1 |
| TCGA-G3-AAUZ-01 | C2 |
| TCGA-G3-A25U-01 | C2 |
| TCGA-DD-AAEK-01 | C2 |
| TCGA-DD-AAEH-01 | C2 |
| TCGA-DD-A73F-01 | C2 |
| TCGA-2Y-A9HA-01 | C1 |
| TCGA-UB-A7MC-01 | C1 |
| TCGA-DD-AACG-01 | C2 |
| TCGA-2Y-A9GY-01 | C1 |
| TCGA-DD-A1EF-01 | C1 |
| TCGA-DD-AADQ-01 | C2 |
| TCGA-DD-AACA-01 | C2 |
| TCGA-DD-A1EK-01 | C2 |
| TCGA-DD-AAD2-01 | C2 |
| TCGA-DD-A11C-01 | C2 |
| TCGA-DD-AAEI-01 | C2 |
| TCGA-G3-AAV5-01 | C1 |
| TCGA-DD-AADK-01 | C2 |
| TCGA-2Y-A9H5-01 | C2 |
| TCGA-FV-A3I0-01 | C1 |
| TCGA-CC-A7IL-01 | C2 |
| TCGA-DD-A115-01 | C2 |
| TCGA-2Y-A9GS-01 | C1 |
| TCGA-ZP-A9CV-01 | C2 |
| TCGA-BC-A69I-01 | C2 |
| TCGA-ZP-A9D1-01 | C2 |
| TCGA-MI-A75C-01 | C2 |
| TCGA-DD-AADV-01 | C2 |
| TCGA-BC-A10Y-01 | C1 |
| TCGA-DD-AACV-01 | C2 |
| TCGA-DD-AADR-01 | C2 |
| TCGA-EP-A2KC-01 | C2 |
| TCGA-DD-AACJ-01 | C2 |
| TCGA-BC-A10R-01 | C2 |
| TCGA-LG-A9QD-01 | C2 |
| TCGA-WX-AA44-01 | C2 |
| TCGA-DD-A113-01 | C2 |
| TCGA-FV-A496-01 | C2 |
| TCGA-DD-A1EC-01 | C2 |
| TCGA-BC-A10Q-01 | C1 |
| TCGA-DD-AAVW-01 | C2 |
| TCGA-2Y-A9H3-01 | C2 |
| TCGA-DD-A4NH-01 | C2 |
| TCGA-CC-A7IE-01 | C2 |
| TCGA-DD-A4NQ-01 | C2 |
| TCGA-DD-A4NG-01 | C2 |
| TCGA-DD-AAW2-01 | C2 |
| TCGA-DD-A73B-01 | C2 |
| TCGA-DD-A4NI-01 | C2 |
| TCGA-MI-A75G-01 | C2 |
| TCGA-BC-A10X-01 | C2 |
| TCGA-CC-A3MC-01 | C2 |
| TCGA-2Y-A9GV-01 | C2 |
| TCGA-DD-A73G-01 | C2 |
| TCGA-CC-5260-01 | C1 |
| TCGA-CC-A7IK-01 | C1 |
| TCGA-DD-A4NL-01 | C2 |
| TCGA-DD-A3A1-01 | C2 |
| TCGA-G3-AAV2-01 | C2 |
| TCGA-BC-A216-01 | C2 |
| TCGA-DD-A4NV-01 | C2 |
| TCGA-ED-A627-01 | C2 |
| TCGA-RC-A7S9-01 | C2 |
| TCGA-DD-AADD-01 | C1 |
| TCGA-BC-A10W-01 | C1 |
| TCGA-BC-A3KG-01 | C1 |
| TCGA-DD-AACN-01 | C1 |
| TCGA-G3-AAV6-01 | C1 |
| TCGA-DD-AAVZ-01 | C2 |
| TCGA-CC-A9FU-01 | C1 |
| TCGA-DD-AACE-01 | C2 |
| TCGA-BC-A3KF-01 | C2 |
| TCGA-ZS-A9CD-01 | C2 |
| TCGA-DD-AADY-01 | C2 |
| TCGA-2Y-A9H4-01 | C2 |
| TCGA-WX-AA47-01 | C2 |
| TCGA-G3-A5SJ-01 | C2 |
| TCGA-YA-A8S7-01 | C1 |
| TCGA-FV-A495-01 | C2 |
| TCGA-DD-A4NP-01 | C2 |
| TCGA-MI-A75I-01 | C1 |
| TCGA-DD-A3A5-01 | C2 |
| TCGA-DD-AACY-01 | C2 |
| TCGA-CC-A5UC-01 | C1 |
| TCGA-CC-A8HV-01 | C2 |
| TCGA-BC-A5W4-01 | C2 |
| TCGA-RC-A7SF-01 | C2 |
| TCGA-RC-A6M4-01 | C2 |
| TCGA-ED-A7XO-01 | C2 |
