# Supplementary material for: Classification of the mitochondrial ribosomal protein-associated molecular subtypes and identified a serological diagnostic biomarker in hepatocellular carcinoma
Source: Front Surg. 2023 Jan 6;9:1062659. doi: 10.3389/fsurg.2022.1062659 (PMC9853988; doi:10.3389/fsurg.2022.1062659)
Supplement: Supplementary file 2 [file Datasheet2.zip › TableS3.docx]

**Table S3** DEGs in two MRPs clusters

| up.genes |  |
| --- | --- |
| TRIP13 | Up |
| CCNB1 | Up |
| HMGA1 | Up |
| KIF2C | Up |
| KIF20A | Up |
| KIFC1 | Up |
| CCNB2 | Up |
| AURKB | Up |
| MYBL2 | Up |
| CDC20 | Up |
| CENPM | Up |
| TPX2 | Up |
| BIRC5 | Up |
| G6PD | Up |
| MCM2 | Up |
| DBN1 | Up |
| TOP2A | Up |
| PTTG1 | Up |
| UBE2C | Up |
| PKM | Up |
| NRSN2 | Up |
| SPHK1 | Up |
| C12orf75 | Up |
| GAL3ST1 | Up |
| ETV4 | Up |
| CA9 | Up |
| DKK1 | Up |
| CD24 | Up |
| SPP1 | Up |
| KRT19 | Up |
| PEG10 | Up |
| AFP | Up |
| EPCAM | Up |
| TAT | Down |
| ACSM2A | Down |
| C8A | Down |
| F9 | Down |
| SLC27A5 | Down |
| SLC22A1 | Down |
| CFHR4 | Down |
| GLYATL1 | Down |
| SLC10A1 | Down |
| PCK1 | Down |
| ALDOB | Down |
| ETNPPL | Down |
| CYP4A22 | Down |
| C6 | Down |
| HP | Down |
| AKR7A3 | Down |
| APOC3 | Down |
| HPX | Down |
| CYP2C8 | Down |
| CYP4F2 | Down |
| ASPDH | Down |
| HRG | Down |
| MOGAT2 | Down |
| CYP2A6 | Down |
| CYP4A11 | Down |
| APOF | Down |
| HPR | Down |
| THRSP | Down |
| SERPINC1 | Down |
| CYP8B1 | Down |
| AGXT | Down |
| AFM | Down |
| FETUB | Down |
| GLYAT | Down |
| UGT2B10 | Down |
| HPD | Down |
| RDH16 | Down |
| RTP3 | Down |
| OTC | Down |
| CFHR3 | Down |
| UROC1 | Down |
| TRIM55 | Down |
| CYP2C9 | Down |
| SLC25A47 | Down |
| ADH1B | Down |
| CCL16 | Down |
| APOA1 | Down |
| CPS1 | Down |
| AQP9 | Down |
| CYP2A7 | Down |
| HSD11B1 | Down |
| HSD17B13 | Down |
| SAA1 | Down |
| SDS | Down |
| HGFAC | Down |
| ADH4 | Down |
| C9 | Down |
| CYP3A4 | Down |
