# Supplementary material for: Classification of the mitochondrial ribosomal protein-associated molecular subtypes and identified a serological diagnostic biomarker in hepatocellular carcinoma
Source: Front Surg. 2023 Jan 6;9:1062659. doi: 10.3389/fsurg.2022.1062659 (PMC9853988; doi:10.3389/fsurg.2022.1062659)
Supplement: Supplementary file 2 [file Datasheet2.zip › TableS4.docx]

**TableS4** Univariate and multivariate analysis based on clinical factors and risk scores

| Characteristics | Total(N) | Univariate analysis | |  | Multivariate analysis | |
| --- | --- | --- | --- | --- | --- | --- |
|  |  | Hazard ratio (95% CI) | P value |  | Hazard ratio (95% CI) | P value |
| Age | 343 | 1.010 (0.996-1.025) | 0.170 |  |  |  |
| Gender | 343 |  |  |  |  |  |
| Female | 109 | Reference |  |  |  |  |
| Male | 234 | 0.772 (0.529-1.127) | 0.180 |  |  |  |
| T | 343 |  |  |  |  |  |
| T1 | 171 | Reference |  |  |  |  |
| T2 | 86 | 1.387 (0.844-2.277) | 0.197 |  | 0.000 (0.000-Inf) | 0.995 |
| T3 | 76 | 2.612 (1.691-4.035) | **<0.001** |  | 1.296 (0.176-9.545) | 0.799 |
| T4 | 10 | 5.074 (2.256-11.411) | **<0.001** |  | 0.281 (0.010-7.911) | 0.456 |
| Stage | 343 |  |  |  |  |  |
| I | 169 | Reference |  |  |  |  |
| II | 84 | 1.433 (0.870-2.358) | 0.157 |  | 4115764.907 (0.000-Inf) | 0.995 |
| III | 85 | 2.731 (1.785-4.177) | **<0.001** |  | 1.495 (0.172-12.991) | 0.715 |
| IV | 5 | 5.585 (1.721-18.127) | **0.004** |  | 2.199 (0.104-46.517) | 0.613 |
| Grade | 343 | 9.646 (1.179-78.931) | **0.035** |  | 14.573 (0.091-2342.887) | 0.301 |
| G1 | 44 | Reference |  |  |  |  |
| G2 | 168 | 2.198 (0.990-4.877) | 0.053 |  | 1.809 (0.799-4.096) | 0.155 |
| G3 | 118 | 3.628 (1.648-7.986) | **0.001** |  | 1.588 (0.510-4.940) | 0.425 |
| G4 | 12 | 9.699 (3.366-27.953) | **<0.001** |  | 11.885 (0.502-281.415) | 0.125 |
| riskScore | 343 | 4.020 (2.690-6.007) | **<0.001** |  | 3.820 (2.011-7.260) | **<0.001** |
