# Supplementary material for: Classification of the mitochondrial ribosomal protein-associated molecular subtypes and identified a serological diagnostic biomarker in hepatocellular carcinoma
Source: Front Surg. 2023 Jan 6;9:1062659. doi: 10.3389/fsurg.2022.1062659 (PMC9853988; doi:10.3389/fsurg.2022.1062659)
Supplement: Supplementary file 2 [file Datasheet2.zip › TableS5.docx]

**TableS6** The diagnostic performances of MRPL9, AFP, and Ferritin in distinguishing HCC from the HC group

| Index | Sensitivity (%) | Specificity (%) | P-value | AUC (95% CI) |
| --- | --- | --- | --- | --- |
| MRPL9 | 76.9 | 93.9 | <0.001 | 0.890 (0.834, 0.945) |
| AFP | 55.1 | 100 | <0.001 | 0.695 (0.603, 0.786) |
| Ferritin | 55.1 | 85.7 | <0.001 | 0.734 (0.648, 0.819) |
| MRPL9+AFP+Ferritin | 85.9 | 98 | <0.001 | 0.958 (0.925, 0.992) |
