# Supplementary material for: Classification of the mitochondrial ribosomal protein-associated molecular subtypes and identified a serological diagnostic biomarker in hepatocellular carcinoma
Source: Front Surg. 2023 Jan 6;9:1062659. doi: 10.3389/fsurg.2022.1062659 (PMC9853988; doi:10.3389/fsurg.2022.1062659)
Supplement: Supplementary file 2 [file Datasheet2.zip › TableS6.docx]

**TableS5** The diagnostic performances of MRPL9, AFP, and Ferritin in distinguishing HCC from the benign group

| Index | Sensitivity (%) | Specificity (%) | P-value | AUC (95% CI) |
| --- | --- | --- | --- | --- |
| MRPL9 | 76.9 | 85 | <0.001 | 0.812 (0.719, 0.905) |
| AFP | 53.8 | 100 | <0.001 | 0.729 (0.632, 0.826) |
| Ferritin | 70.5 | 75 | <0.001 | 0.757 (0.655, 0.860) |
| MRPL9+AFP+Ferritin | 78.2 | 100 | <0.001 | 0.933 (0.884, 0.981) |
