# Supplementary material for: Classification of the mitochondrial ribosomal protein-associated molecular subtypes and identified a serological diagnostic biomarker in hepatocellular carcinoma
Source: Front Surg. 2023 Jan 6;9:1062659. doi: 10.3389/fsurg.2022.1062659 (PMC9853988; doi:10.3389/fsurg.2022.1062659)
Supplement: Supplementary file 2 [file Datasheet2.zip › TableS7.docx]

**TableS7** The diagnostic performances of MRPL9, AFP, and Ferritin in distinguishing HCC from the benign and HC groups

| Index | Sensitivity (%) | Specificity (%) | P-value | AUC (95% CI) |
| --- | --- | --- | --- | --- |
| MRPL9 | 76.9 | 91.3 | <0.001 | 0.867 (0.807, 0.927) |
| AFP | 53.8 | 100 | <0.001 | 0.705 (0.616, 0.793) |
| Ferritin | 43.6 | 97.1 | <0.001 | 0.740 (0.661, 0.820) |
| MRPL9+AFP+Ferritin | 85.9 | 92.8 | <0.001 | 0.948 (0.913, 0.983) |
